# Age‐induced mitochondrial DNA point mutations are inadequate to alter metabolic homeostasis in response to nutrient challenge

**DOI:** 10.1111/acel.13166

**Published:** 2020-10-13

**Authors:** Timothy M. Moore, Zhenqi Zhou, Alexander R. Strumwasser, Whitaker Cohn, Amanda J. Lin, Kevin Cory, Kate Whitney, Theodore Ho, Timothy Ho, Joseph L. Lee, Daniel H. Rucker, Austin N. Hoang, Kevin Widjaja, Aaron D. Abrishami, Sarada Charugundla, Linsey Stiles, Julian P. Whitelegge, Lorraine P. Turcotte, Jonathan Wanagat, Andrea L. Hevener

**Affiliations:** ^1^ Department of Biological Sciences Dana & David Dornsife College of Letters, Arts, and Sciences University of Southern California Los Angeles CA USA; ^2^ Division of Endocrinology, Diabetes, and Hypertension Department of Medicine David Geffen School of Medicine University of California Los Angeles CA USA; ^3^ Department of Psychiatry and Biobehavioral Sciences & The Semel Institute for Neuroscience and Human Behavior University of California Los Angeles CA USA; ^4^ Division of Cardiology Department of Medicine David Geffen School of Medicine University of California Los Angeles CA USA; ^5^ Iris Cantor‐UCLA Women's Health Center University of California Los Angeles CA USA

**Keywords:** aging, insulin resistance, metabolism, mitochondria, mitochondrial DNA, obesity, POLG

## Abstract

Mitochondrial dysfunction is frequently associated with impairment in metabolic homeostasis and insulin action, and is thought to underlie cellular aging. However, it is unclear whether mitochondrial dysfunction is a cause or consequence of insulin resistance in humans. To determine the impact of intrinsic mitochondrial dysfunction on metabolism and insulin action, we performed comprehensive metabolic phenotyping of the polymerase gamma (PolG) D257A “mutator” mouse, a model known to accumulate supraphysiological mitochondrial DNA (mtDNA) point mutations. We utilized the heterozygous PolG mutator mouse (PolG^+/mut^) because it accumulates mtDNA point mutations ~ 500‐fold > wild‐type mice (WT), but fails to develop an overt progeria phenotype, unlike PolG^mut/mut^ animals. To determine whether mtDNA point mutations induce metabolic dysfunction, we examined male PolG^+/mut^ mice at 6 and 12 months of age during normal chow feeding, after 24‐hr starvation, and following high‐fat diet (HFD) feeding. No marked differences were observed in glucose homeostasis, adiposity, protein/gene markers of metabolism, or oxygen consumption in muscle between WT and PolG^+/mut^ mice during any of the conditions or ages studied. However, proteomic analyses performed on isolated mitochondria from 12‐month‐old PolG^+/mut^ mouse muscle revealed alterations in the expression of mitochondrial ribosomal proteins, electron transport chain components, and oxidative stress‐related factors compared with WT. These findings suggest that mtDNA point mutations at levels observed in mammalian aging are insufficient to disrupt metabolic homeostasis and insulin action in male mice.

## Graphical abstract

Mice harboring supraphysiological mtDNA point mutations (PolG^+/mut^) respond similarly to WT animals during metabolic challenge including nutrient excess, starvation, and acute exercise.

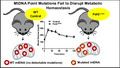

**Abbreviations**
4EBP1: Eukaryotic Translation Initiation Factor 4E‐Binding Protein 1
18S: 18S Ribosomal RNA
Abcb10: ATP Binding Cassette Subfamily B Member 10
ACACA: Acetyl‐CoA Carboxylase Alpha
ACACB: Acetyl‐CoA Carboxylase Beta
ACADL: Acyl‐CoA Dehydrogenase, Long Chain
ACADM: Acyl‐CoA Dehydrogenase, Medium Chain
ACC: Acetyl‐CoA Carboxylase Alpha
ACOX1: Acyl‐CoA Oxidase 1
ACSL1: Acyl‐CoA Synthetase Long‐Chain Family Member 1
AMPKa: Protein Kinase AMPK‐Activated Catalytic Subunit Alpha 1
Atg10: Autophagy‐Related 10
Atg12: Autophagy‐Related 12
Atg3: Autophagy‐Related 3
Atg5: Autophagy‐Related 5
Atg7: Autophagy‐Related 7
ATGL: Adipose Triglyceride Lipase
BAT: Brown Adipose Tissue
C/EBPa: CCAAT/Enhancer‐Binding Protein Alpha
CD36: Cluster Determinant 36 Molecule
C‐I‐20: Mitochondrial Complex 1 NADH:Ubiquinone Oxidoreductase Subunit B8
C‐II‐30: Mitochondrial Complex 2 Succinate Dehydrogenase Complex Iron–Sulfur Subunit B
C‐III‐Core 2: Mitochondrial Complex 3 Ubiquinol‐Cytochrome C Reductase Core Protein II
C‐IV‐I: Mitochondrial Encoded Cytochrome C Oxidase I
CO1: Mitochondrial Encoded Cytochrome C Oxidase I
CO2: Mitochondrial Encoded Cytochrome C Oxidase II
CO3: Mitochondrial Encoded Cytochrome C Oxidase III
COX: Cytochrome C Oxidase
Cpt1b: Carnitine Palmitoyltransferase 1B
C‐V‐a: Mitochondrial Complex 5 ATP Synthase Alpha
Cyto: Cytoplasmic
DGAT1: Diacylglycerol O‐Acyltransferase 1
DGAT2: Diacylglycerol O‐Acyltransferase 2
DJ1: Parkinson Disease 7
Drp1: Dynamin‐Related Protein 1
eWAT: Epidydimal White Adipose Tissue
FABP4: Fatty Acid‐Binding Protein 4
FASN: Fatty Acid Synthase
FGF21: Fibroblast Growth Factor 21
FOXO1: Forkhead Box O1
GAPDH: Glyceraldehyde‐3‐Phosphate Dehydrogenase
Gfm2: G Elongation Factor Mitochondrial 2
GLUT4: Glucose Transporter Type 4
GPAM: Glycerol‐3‐Phosphate Acyltransferase, Mitochondrial
GPX3: Glutathione Peroxidase 3
HFD: High‐Fat Diet
HKII: Hexokinase 2
HSP60: Heat Shock Protein Family D Member 1
HSPA9: Heat Shock Protein Family A Member 9
HSPD1: Heat Shock Protein Family D Member 1
HSPE1: Heat Shock Protein Family E Member 1
IFNg: Interferon Gamma
IL‐10: Interleukin‐10
IL‐6: Interleukin‐6
IRS1: Insulin Receptor Substrate 1
iWAT: Inguinal White Adipose Tissue
Jmjd3: Jumonji Domain‐Containing Protein 3
Lpin1a: Lipin 1a
Lpin1b: Lipin 1b
LPL: Lipoprotein Lipase
MAPLC3B: Microtubule‐Associated Protein 1 Light Chain 3 Beta
MFF: Mitochondrial Fission Factor
MFN1: Mitofusin 1
MFN2: Mitofusin 2
Mgme1: Mitochondrial Genome Maintenance Exonuclease 1
Mid51: Mitochondrial Dynamics Protein of 51 kDa
Mito: Mitochondrial
mtDNA: Mitochondrial DNA
ND1: Mitochondrial Encoded NADH:Ubiquinone Oxidoreductase Core Subunit 1
ND2: Mitochondrial Encoded NADH:Ubiquinone Oxidoreductase Core Subunit 2
ND4: Mitochondrial Encoded NADH:Ubiquinone Oxidoreductase Core Subunit 4
ND4L: Mitochondrial Encoded NADH:Ubiquinone Oxidoreductase Core Subunit 4L
ND5: Mitochondrial Encoded NADH:Ubiquinone Oxidoreductase Core Subunit 5
ND6: Mitochondrial Encoded NADH:Ubiquinone Oxidoreductase Core Subunit 6
NRF1: Nuclear Respiratory Factor 1
Nuc: Nuclear
Opa1: Optic Atrophy Protein 1
Park2: Parkin RBR E3 Ubiquitin Protein Ligase
Peo1: Twinkle mtDNA Helicase
PGC1a: PPARG Coactivator 1 Alpha
Pink1: PTEN‐Induced Putative Kinase 1
Plin1: Perilipin 1
PolG: DNA Polymerase Gamma, Catalytic Subunit
PolG2: DNA Polymerase Gamma 2, Accessory Subunit
Polrmt: RNA Polymerase Mitochondrial
PPARg: Peroxisome Proliferator‐Activated Receptor Gamma
Quad: Quadriceps
Satb1: SATB Homeobox 1
SOD2: Superoxide Dismutase 2
SQSTM1: Sequestosome 1
TFAM: Transcription Factor A, Mitochondrial
TNF‐a: Tumor Necrosis Factor Alpha
TXN1: Thioredoxin 1
TXN2: Thioredoxin 2
TXNIP: Thioredoxin Interacting Protein
TXNRD1: Thioredoxin Reductase 1
Ulk1: Unc‐51 Like Autophagy‐Activating Kinase 1

## 1 INTRODUCTION

Mitochondria are essential for respiration and the regulation of diverse cellular processes; thus, mitochondrial dysfunction is believed to underlie a variety of metabolic and aging‐related diseases (Bogacka, Xie, Bray, & Smith, 2005; Center; Hesselink, Schrauwen‐Hinderling, & Schrauwen, 2016; Joseph, Joanisse, Baillot, & Hood, 2012; Montgomery & Turner, 2015; Petersen et al., 2003; Wanagat & Hevener, 2016; Wang et al., 2013; Yuzefovych, Musiyenko, Wilson, & Rachek, 2013; Zabielski et al., 2016). Mutations in the mitochondrial genome are thought to drive mitochondrial dysfunction and have been implicated in aging‐related diseases; however, whether mtDNA mutations are causal or consequent of metabolic dysfunction remains unclear (Avital et al., 2012; Gilkerson, 2016; Hicks et al., 2013; Lowell & Shulman, 2005; Monickaraj et al., 2012; Nile et al., 2014; Nomiyama et al., 2002; Tranah et al., 2011; Wallace, 2015; Wang et al., 2013). The polymerase gamma (PolG) “mutator” mouse is a model of intrinsic mitochondrial dysfunction and was employed for this study to determine whether mtDNA mutations are sufficient to drive metabolic abnormalities and aging‐associated insulin resistance and adiposity (Kujoth et al., 2005; Trifunovic et al., 2004).

Mice harboring a homozygous PolG loss of proofreading 3′‐5′ exonuclease function mutation (PolG^mut/mut^) develop mtDNA point mutations at a rate that far exceeds mutations observed in aged wild‐type (WT) animals and humans (Herbst et al., 2016, 2017; Vermulst et al., 2007). The mtDNA point mutations that accumulate in young PolG^mut/mut^ mice (~136‐fold increase versus WT mice) manifest a variety of preadolescent phenotypic abnormalities including progeroid‐like symptoms throughout maturation as well as premature death (~12–16 months of age) (Chen et al., 2010; Fox, Magness, Kujoth, Prolla, & Maeda, 2012; Jin & Youle, 2012; Joseph et al., 2013; Kujoth et al., 2005; Ross, Coppotelli, Hoffer, & Olson, 2014; Safdar et al., 2011; Saleem et al., 2015; Seo et al., 2010; Trifunovic et al., 2004; Vermulst et al., 2007). Because of the complexity of the early‐onset aging, we studied the PolG heterozygous (PolG^+/mut^) mouse, which lacks progeroid‐like symptoms despite a supraphysiological mtDNA point mutation frequency (~30‐fold ↑ mutation load in PolG^+/mut^ versus WT mice) (Trifunovic et al., 2004; Vermulst et al., 2007). Furthermore, male and female PolG^+/mut^ mice show no significant difference in lifespan versus WT animals (tested up to 800 days of age) (Kujoth et al., 2005).

Based on previous reports, we hypothesized that an increased mtDNA point mutation frequency in PolG^+/mut^ mice would promote mitochondrial dysfunction and accelerate the development of insulin resistance during aging. We examined specific aspects of metabolism in male PolG^+/mut^ mice at 6 and 12 months of age under three dietary conditions: normal chow (NC) feeding, high‐fat feeding (HFD), and 24‐hr starvation. We performed mitochondrial proteomics and assessed dynamics and quality control signaling in muscle and liver to determine whether mitochondria respond to mtDNA point mutations by altering morphology and turnover. In the current study, we observed that the accumulation of mtDNA point mutations failed to disrupt metabolic homeostasis and insulin action in male mice, but with aging, metabolic health was likely preserved by countermeasures against oxidative stress and compensation by the mitochondrial proteome.

## 2 RESULTS

### 2.1 mtDNA point mutations fail to disrupt glucose homeostasis, plasma metabolites, and exercise capacity

**Figure 1:**
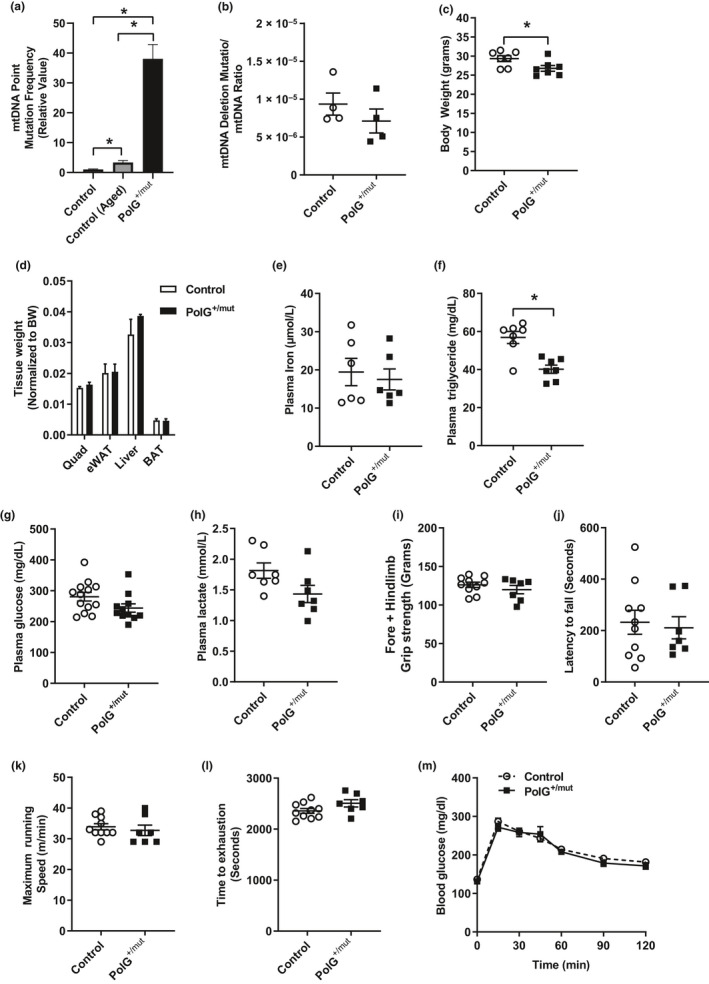
Increased mtDNA point mutations do not induce insulin resistance or adiposity in male mice at 6 months of age. Mitochondrial DNA (a) point mutation frequency and (b) deletion mutation frequency in muscle from PolG^+/mut^ versus Control. (c) Body weight and (d) wet tissue weight in Control and PolG^+/mut^ male mice. (e–h) Iron, triglyceride, lactate, and glucose levels measured in plasma from Control and PolG^+/mut^ following a 6‐hr fast. (i–l) Muscle grip strength, latency to fall, maximum running speed, and time to running exhaustion. (m) Glucose tolerance test. Values are expressed as means ± *SEM*, and mean differences were detected by Student's *t* test. *p < .05 between‐group significance, PolG^+/mut^ versus Control. *N* = 6–11/group

We quantified both mtDNA point and deletion mutations in mouse quadriceps muscle using the highly sensitive droplet digital PCR method and detected a robust increase in mtDNA point mutations, but no change in mtDNA deletion mutations in PolG^+/mut^ versus WT control (Figure 1a,b, *p* < .05). These findings are congruent with previous reports by Vermulst et al. (2007, 2008). Next, we performed a comprehensive phenotypic evaluation of male PolG^+/mut^ mice under a variety of metabolic perturbations to understand the impact of mtDNA point mutations on whole‐body metabolism. During normal chow feeding, PolG^+/mut^ mice (6 months of age) displayed a significantly lower body weight (8.7% reduction) compared withWT control littermates (Figure 1c, *p* < .05). Quadriceps muscle (Quad), epididymal white adipose tissue (eWAT), liver, and brown adipose tissue (BAT) weights were not different between the two groups of mice when normalized to total body weight (Figure 1d, *p* > .05).

Although anemia was observed in the PolG^mut/mut^ “mutator” mouse (Ahlqvist et al., 2015), plasma iron levels were not different between PolG^+/mut^ and littermate control mice (Figure 1e, *p* > .05). Plasma triglyceride concentration was reduced by 29.3% in PolG^+/mut^ (Figure 1f, *p* < .05; 6‐hr fasted state), but no difference in plasma glucose and lactate concentrations was detected between the groups (Figure 1g–h, *p* > .05). To determine the impact of mtDNA point mutations on muscle function, strength, and exercise capacity, mice performed tests of grip strength, latency to fall (dynamic hanging test), running speed, and ‐running endurance/time to exhaustion. No differences were observed for muscle strength and exercise capacity (maximum running speed or time to exhaustion) in PolG^+/mut^ mice versus littermate controls at 6 months of age (Figure 1i–l, *p* > .05). Glucose tolerance was also identical between the groups (Figure 1m, *p* > .05). Our data show no overt impact of mtDNA point mutations on fasting blood glucose, glucose tolerance, adiposity, or muscle strength and endurance exercise capacity in 6‐month‐old male mice fed a normal chow diet.

### 2.2 mtDNA point mutations fail to impact muscle mitochondrial function

**Figure 2:**
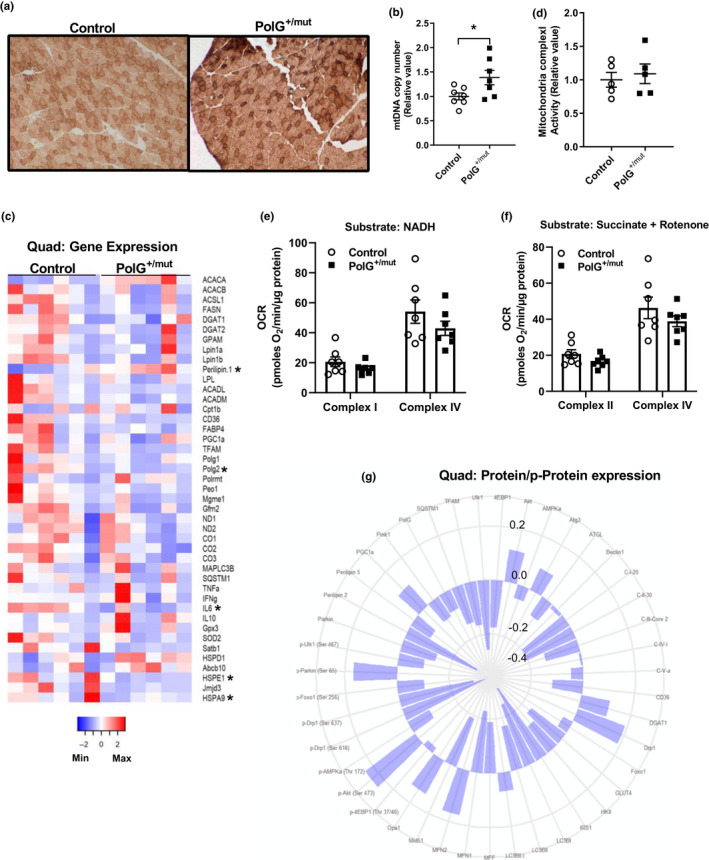
Increased mtDNA point mutations in muscle increase mtDNA copy number. (a) Cytochrome C oxidase (COX)‐positive fibers (darker brown) in muscle sections from the tibialis anterior with representative images from each group. (b) Mitochondrial DNA copy number in quadriceps muscle of PolG^+/mut^ versus normalized Control. (c) Heat map of mRNA expression in quadriceps muscles normalized to Control. (d) Mitochondrial complex 1 activity in gastrocnemius muscle and normalized to Control. (e–f) Oxygen consumption rates from complex I and complex IV in quadriceps muscle expressed as picomoles of oxygen/minute/ microgram of protein using NADH or succinate + rotenone as substrates. (g) Polar histogram of fold change in protein/phosphoprotein expression in whole quadriceps muscle lysates of PolG^+/mut^ over Control. Protein name depicted at edge with each successive circle representing a 0.2‐, 0.0‐, ‐0.2‐, or ‐0.4‐fold change compared with Control. Values are expressed as means ± *SEM*, and mean differences were detected by Student's *t* test. *p < .05, significantly different from Control. *N* = 4–7/group

To determine the impact of mtDNA point mutations on mitochondrial function, we assessed gene and protein expression, enzymatic activity, and respiration in skeletal muscle of 6‐month‐old male PolG^+/mut^ mice. We studied skeletal muscle because of its central role in maintaining whole‐body metabolism and insulin sensitivity. First, histological analyses showed no gross morphological abnormalities and no difference in cytochrome c oxidase staining between PolG^+/mut^ and control mouse muscle (Figure 2a).

Interestingly, the robust accumulation of mtDNA point mutations drove a 39% increase in mtDNA copy number (Figure 2b, *p* < .05), although this increase in quadriceps muscle CN failed to elevate expression of mitochondrial encoded genes, for example, ND1, ND2, CO1, CO2, and CO3 (Figure 2c). Furthermore, we detected no difference in mitochondrial complex I activity (Figure 2d), or oxygen consumption rate for complex I and complex IV (using NADH and succinate + rotenone as substrates) in quadriceps muscle of PolG^+/mut^ mice versus WT controls (Figure 2e–f, *p* > .05).

Expression levels of quadriceps muscle genes associated with fatty acid metabolism, mitochondrial function, autophagy, inflammation, and the unfolded protein response were identical between the groups (Figure 2c). We did, however, observe differential expression of *ACACA*, *Perilipin 1*, *Polg2* (encodes the accessory subunit of the PolG enzyme complex), *IL6*, and the genes encoding the heat shock proteins Hsp10 and cognate Hsp70, that is, *HSPE1* and *HSPA9,* respectively*,* in muscle from PolG^+/mut^ mice compared with WT (Figure 2c, *p* < .05). Protein signaling related to insulin action, protein synthesis, energy homeostasis, lipid metabolism, autophagy, mitochondrial dynamics, and the electron transport chain was also similar between the groups (Figure 2g, Figure S1, *p* > .05). Overall, despite the marked increase in mtDNA point mutations in muscle of male PolG^+/mut^ mice (6 months of age), no change in mRNA and protein/p‐protein expression levels, respiration, or mitochondrial complex I enzyme activity was observed in PolG^+/mut^ mice compared with control WT littermates.

### 2.3 mtDNA point mutations fail to disrupt gene or protein expression, mtDNA copy number, or metabolite levels in liver

Since liver is also rich in mitochondria and is an organ critical for metabolic homeostasis, we studied the impact of mtDNA point mutations in 6‐month‐old male PolG^+/mut^ mice. Similar to muscle, mtDNA point mutations in liver were dramatically increased in PolG^+/mut^ mice compared with WT control animals (Figure S2a, *p* < .05). However, in contrast to muscle, hepatic mtDNA copy number was identical between the groups (Figure S2b, *p* > .05). Overall hepatic expression of genes involved in fatty acid metabolism was unaffected by mtDNA point mutations, although we did detect modest yet significant differential expression of three genes, *ACACB*, *FASN*, and *CD36*, in normal chow‐fed PolG^+/mut^ versus littermate controls (Figure S2c, *p* < .05). Moreover, no differences in bulk expression of proteins associated with autophagy, mitochondrial dynamics, electron transport chain, fatty acid metabolism, glucose metabolism, and insulin signaling were detected between 6‐month‐old male PolG^+/mut^ mice compared with controls (Figure S2d, *p* < .05). In line with gene expression, liver glycogen concentration (Figure S2e, *p* > .05) and hepatic lipids (triglyceride, TG; total cholesterol, TC; unesterified cholesterol, UC; phosphatidylcholine, PC; and cholesterol ester, CE) were identical between the groups (Figure S2f, p > .05).

### 2.4 mtDNA point mutations fail to impact response to 24‐hr starvation stress

**Figure 3:**
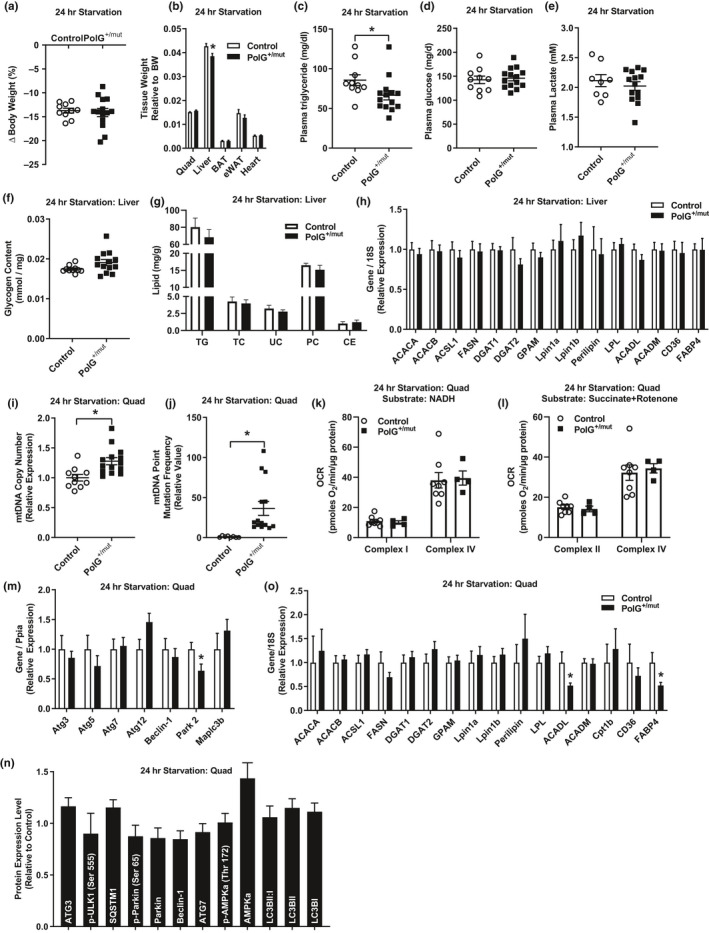
Increased mtDNA point mutations do not alter metabolic response to 24‐hr starvation. (a) Change in body weight and (b) wet tissue weights in PolG^+/mut^ versus Control following 24‐hr starvation. (c–e) Plasma triglycerides, glucose, and lactate levels. (f) Hepatic glycogen content expressed relative to g of tissue. (g) Hepatic lipid concentration expressed as milligram of lipid/ gram of liver (TG, triglyceride; TC, total cholesterol; UC, unesterified cholesterol; PC, phosphatidylcholine; CE, cholesterol ester). (h) Hepatic gene expression relative to Control normalized to 1.0. (i) Mitochondrial DNA (i) point mutation frequency and (j) copy number in quadriceps muscles from PolG^+/mut^ versus normalized Control. (k–l) Measurement of oxygen consumption rate from complex I and complex IV in quadriceps muscle expressed as picomoles of oxygen/ minute/ microgram of protein with either NADH or succinate + rotenone as substrates. (m–n) Gene and protein/phosphoprotein expression of mitophagy markers in quadriceps muscle from PolG^+/mut^ relative to normalized Control following 24‐hr starvation. (o) Expression of genes regulating lipid metabolism in PolG^+/mut^ relative to normalized Control. Values are expressed as means ± *SEM*, and mean differences were detected by Student's *t* test. *p < .05, significantly different from Control. *N* = 8–14/group

Next, we challenged PolG^+/mut^ mice with 24‐hr starvation to determine whether excessive mtDNA point mutation frequency impacted metabolic adaptation to nutrient deprivation. Starvation induced a comparable 14% reduction in total body weight for both PolG^+/mut^ and littermate control mice (Figure 3a, *p* > .05). Quadriceps muscle, BAT, eWAT, and heart weights (relative to body weight) were also comparable between the groups, although the reduction in liver weight was significantly greater in PolG^+/mut^ mice compared with control (9.8% reduction, Figure 3b, *p < .05*). Nutrient deprivation reduced plasma triglyceride concentration to a greater extent in PolG^+/mut^ versus control mice (Figure 3c, *p* < .05), whereas glucose and lactate levels were not different between groups (Figure 3d,e, *p* > .05), findings similar to those obtained in age‐matched postabsorptive animals.

Despite the reduced liver weight in PolG^+/mut^ mice after 24‐hr starvation, hepatic glycogen and lipid content was not statistically different between the two experimental groups (Figure 3f,g, *p* > .05). The expression of hepatic genes associated with fatty acid metabolism was also not different between the groups (Figure 3h, *p* > .05).

Similar to muscle in the postabsorptive state, following 24 hr of starvation both mtDNA point mutation frequency and mtDNA copy number were elevated in quadriceps muscle of PolG^+/mut^ versus control (Figure 3i,j, *p* < .05). Despite the increase in mtDNA copy number, mitochondrial complex I and complex IV oxygen consumption rates (using NADH and succinate + rotenone as substrates) were identical in quadriceps muscle of PolG^+/mut^ mice versus littermate controls (Figure 3k,l, *p* > .05). We examined gene expression and protein abundance of markers associated with macro‐ and micro‐autophagy and fatty acid metabolism, and although most of the markers were not different between the groups, we did observe that *Park2*, *ACADL*, and *FABP4* transcripts were reduced in PolG^+/mut^ quadriceps muscle compared with control animals (Figure 3m–o, Figure S3, *p* < .05). In aggregate, these data indicate that at 6 months of age, mtDNA point mutations fail to impact metabolic responsiveness of male mice to 24‐hr caloric deprivation.

### 2.5 mtDNA point mutations fail to impact high‐fat diet‐induced effects on muscle metabolism

**Figure 4:**
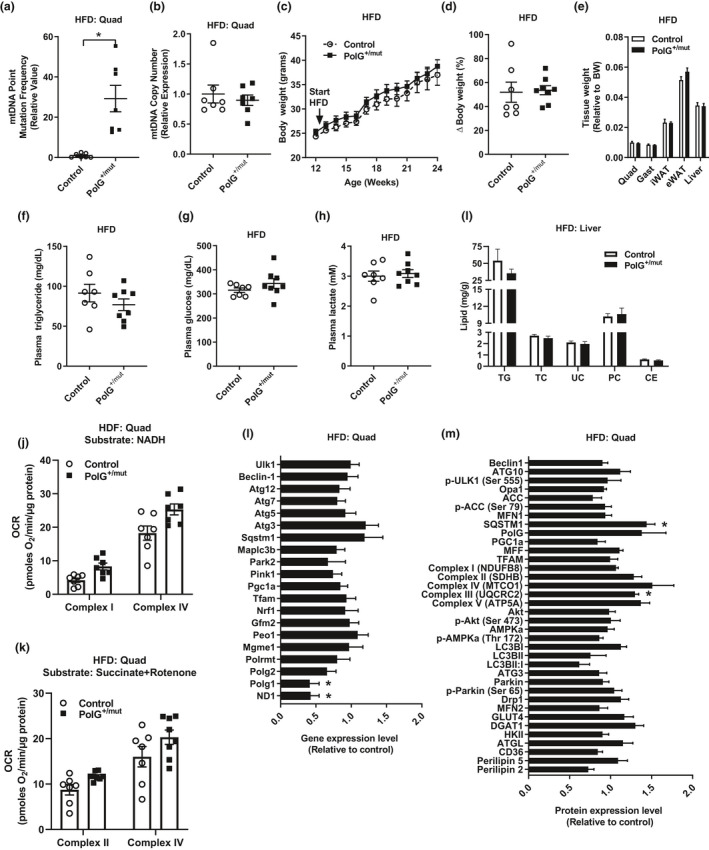
Increased mtDNA point mutations do not alter metabolic response to HFD feeding. Mitochondrial DNA (a) point mutation frequency and (b) DNA copy number in quadriceps muscles from PolG^+/mut^ relative to normalized Control. (c) Weekly body weight in mice after initiation of HFD at 12 weeks of age. (d) Change in body weight between groups consuming a HFD. (e) Wet tissue weight relative to body weight. (f–h) Plasma triglyceride, glucose, and lactate. (i) Hepatic lipid concentration expressed as milligram of lipid/gram of liver (TG, triglyceride; TC, total cholesterol; UC, unesterified cholesterol; PC, phosphatidylcholine; CE, cholesterol ester). (j–k) Oxygen consumption rates from complex I and complex IV in quadriceps muscle expressed as picomoles of oxygen/minute/microgram of protein using NADH or succinate + rotenone as substrates. Quadriceps muscle (l) gene and (m) protein/phosphoprotein expression in HFD‐fed PolG^+/mut^ versus normalized Control. Values are expressed as means ± *SEM*, and mean differences were detected by Student's *t* test. *p < .05, significantly different from Control. *N* = 6–8/group

Next, we determined whether elevated mtDNA point mutations exacerbated high‐fat diet feeding disruption in metabolism. Similar to postabsorptive and 24‐hr starved animals, a markedly elevated mtDNA point mutation load was maintained in muscle from HFD‐fed PolG^+/mut^ mice compared with control animals (Figure 4a, *p* < .05). However, in contrast to normal chow‐fed animals, muscle mtDNA copy number was not different between the groups after 12 weeks of consuming a HFD (Figure 4b, *p* > .05). Weight gain during consumption of the HFD was similar between male PolG^+/mut^ and littermate control mice (Figure 4c,d, *p* > .05), as were muscle (Quad and Gast), white adipose tissue (iWAT and eWAT), and liver weights (Figure 4e, *p* > .05). Moreover, plasma triglycerides, glucose, and lactate concentrations, as well as hepatic lipid levels, were identical between PolG^+/mut^ and WT animals (Figure 4f–i, *p* > .05).

Similar to previous observations for muscle of normal chow‐fed mice, mitochondrial complex I and complex IV oxygen consumption rates were not different between the two groups of HFD‐fed mice (Figure 4j–k, *p* > .05). Moreover, gene expression and protein expression of factors associated with mitochondrial dynamics, electron transport chain, autophagy, fatty acid, and glucose metabolism were identical between the two groups following HFD feeding (Figure 4l–m, Figure S4, *p* > .05), with only a few genes/proteins differentially expressed between the groups. Taken together, our findings show that a higher mtDNA point mutation load in male PolG^+/mut^ mice fails to impact the response to nutrient deprivation and excess.

### 2.6 mtDNA point mutations fail to accelerate characteristic features of aging

**Figure 5:**
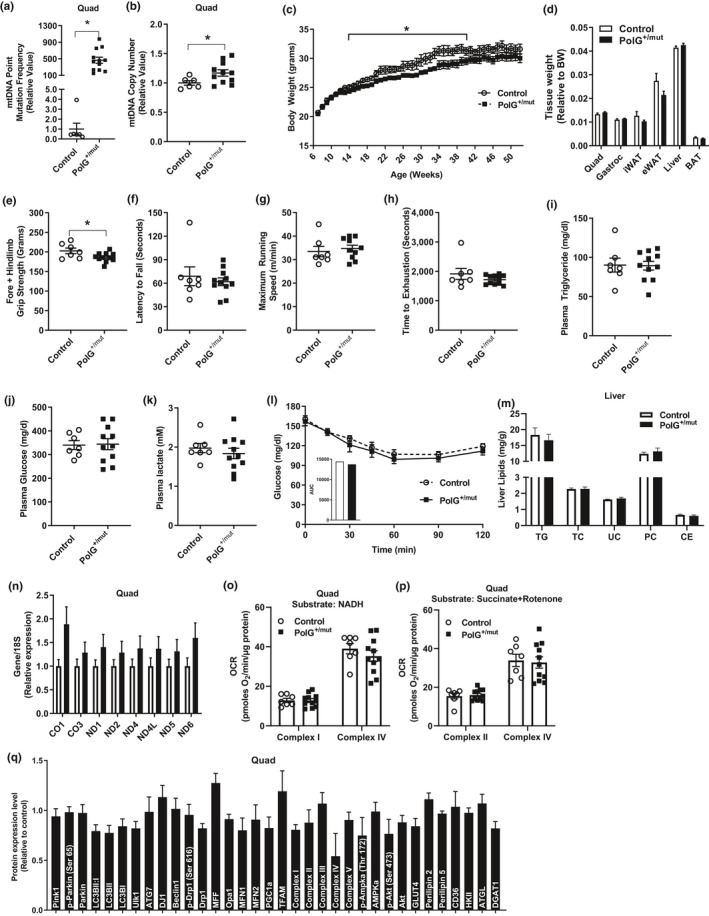
Increased mtDNA point mutations fail to disrupt insulin sensitivity or promote adiposity in aged mice. Mitochondrial DNA (a) point mutation frequency and (b) DNA copy number in quadriceps muscle from PolG^+/mut^ relative to normalized Control. (c) Weekly body weight in aging mice. (d) Wet tissue weights relative to body weight of PolG^+/mut^ versus Control. (e–h) Muscle strength and endurance. (i–k) Plasma triglyceride, glucose, and lactate concentrations. (l) Insulin tolerance test with area under the curve (AUC) insert. (m) Hepatic lipid levels expressed as milligram of lipid/gram of liver (TG, triglyceride; TC, total cholesterol; UC, unesterified cholesterol; PC, phosphatidylcholine; CE, cholesterol ester). (n) Muscle expression of genes encoded by the mitochondrial genome. (o–p) Muscle oxygen consumption rates from complex I and complex IV expressed as picomoles of oxygen/minute/microgram of quadriceps protein using NADH or succinate + rotenone as substrates. (q) Expression of proteins/phosphoproteins in muscle from PolG^+/mut^ relative to Control. Values are expressed as means ± *SEM*. Mean differences were detected by Student's *t* test. *p < .05, significantly different from Control. *N* = 8–15/group

Since PolG^+/mut^ mice accumulate mtDNA point mutations sporadically with age, we reasoned that aging may drive greater disruption of metabolic homeostasis in PolG^+/mut^ mice versus control. Similar to younger cohorts of animals, muscle mtDNA point mutation frequency (Figure 5a, *p* < .05) and copy number Figure 5b, *p* < .05) were elevated in PolG^+/mut^ mice compared with WT littermate controls. Body weight was reduced from 14–52 weeks of age for male PolG^+/mut^ mice compared with littermate controls, but only reached statistical significance between the groups from 16 to 40 weeks of age (Figure 5c, *p* < .05). At 12 months of age, quad, gast, iWAT, eWAT, liver, and BAT weights were similar between the two groups when expressed relative to body weight (Figure 5d, *p* > .05), and these findings were similar to observations in younger mouse cohorts.

To assess functional capacity of aged animals, grip strength, latency to fall (dynamic hanging), maximum running speed, and endurance exercise capacity tests (time to exhaustion) were performed by animals at 12 months. Grip strength was 8.0% lower in PolG^+/mut^ mice versus control, while there were no differences detected between the groups for any of the other functional tests (Figure 5e–h, *p* < .05). Plasma triglyceride, glucose, and lactate concentrations were similar between aged PolG^+/mut^ versus control WT mice following a 6‐hr fast (Figure 5i–k, *p* > .05). Insulin sensitivity (assessed by insulin tolerance test) and hepatic lipid levels were also similar between the two groups of aged mice (Figure 5l–m, *p* > .05).

The increase in mtDNA copy number in quadriceps muscle was paralleled by increased expression levels of eight mitochondrial encoded genes, although none reached statistical significance (Figure 5n, *p* > .05). However, despite the increase in mtDNA CN, oxygen consumption was identical between muscle samples for the aged groups of mice independent of substrate provided (Figure 5o‐p, *p* < .05). Moreover, protein expression of regulators of mitochondrial quality control and muscle metabolism were also not different between groups (Figure 5q, Figure S5, *p > .05*). Thus, our findings indicate that mtDNA point mutations do not accelerate the emergence of traditional metabolic characteristics of aging in male mice.

### 2.7 mtDNA point mutations alter the mitochondrial proteome in muscle from aged mice

**Figure 6:**
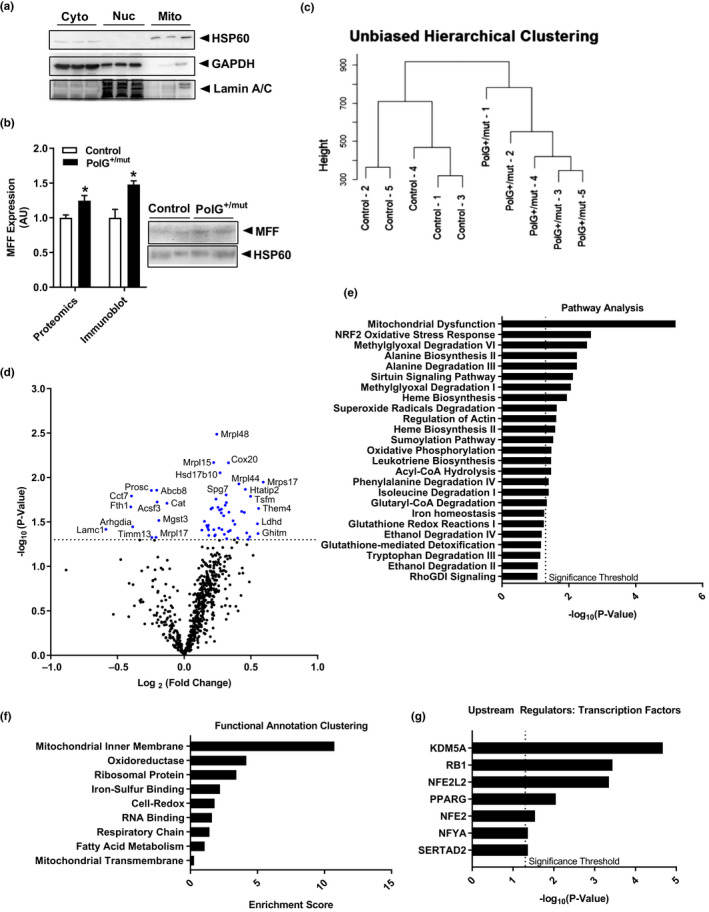
Increased mtDNA point mutations alter the mitochondrial proteome of muscle from aged mice. (a) Representative immunoblot verifying cytosolic (Cyto), nuclear (Nuc), and mitochondrial (Mito) fractions of control muscle. (b) Selected mitochondrial protein, fission factor MFF, to corroborate findings from a mitochondrial proteomic screen compared with immunoblotting. (c) Unbiased hierarchical clustering of mice indicating genotype and sample #. (d) Volcano plot of proteins identified in the muscle mitochondrial proteomic screen expressed as fold change for 12‐month‐old PolG^+/mut^ relative to Control (dotted line indicates significance threshold, and blue dots indicate proteins significantly altered in PolG^+/mut^ relative to control). (e) Pathway analysis of significantly altered proteins in muscle from aged PolG^+/mut^ relative to Control (dotted line indicates significance threshold). (f) Functional annotation clustering of significantly altered proteins identified in mitochondrial proteomic screen expressed as an enrichment score. (g) Transcription factor association of significantly altered proteins expressed as significance of association from TRRUST analysis (dotted line indicates significance threshold). * *p* < .05 significantly different from Control. *N* = 5/group

To further interrogate the impact of mtDNA point mutations on mitochondrial biology, we performed proteomic analyses on mitochondria isolated from gastrocnemius muscle of 12‐month‐old male PolG^+/mut^ and littermate control mice (Figure 6a). Our analyses showed strong reproducibility of select mitochondrial proteins between mass spectrometry versus immunoblotting (Figure 6b). Hierarchical cluster analysis of the mitochondrial proteome distinctly segregated the mice by genotype, indicating that PolG^+/mut^ possess a distinct proteomic phenotype compared with control animals (Figure 6c). Of the peptides detected by mass spectrometry, 73% were differentially expressed in PolG^+/mut^ male mice compared with controls; however, only 58 in total (~9%) reached statistical significance (Table S3, *p < .05*; Figure 6d).

Pathway analysis of significantly increased proteins highlighted alterations in mitochondrial function, NRF2 oxidative stress, sirtuin signaling, and heme metabolism (Figure 6e, *p < .05*). Functional annotation clustering of significantly impacted proteins also revealed associations with the mitochondrial inner membrane, oxidative stress, ribosomal proteins, and heme regulation (Figure 6f). Of note, 84% of proteins involved in the electron transport chain were elevated in PolG^+/mut^ over control, although only six reached statistical significance.

Lastly, Transcriptional Regulatory Relationships Unraveled by Sentence‐based Text mining (TRRUST) analysis predicted histone demethylase Kdm5a, retinoblastoma (Rb1), and NFE2/Nrf2 which collectively regulate muscle metabolism (Uruno et al., 2016), myogenic differentiation (Zappia, Rogers, Islam, & Frolov, 2019), mitobiogenesis (Crilly, Tryon, Erlich, & Hood, 2016; Hu et al., 2018; Huang et al., 2019), and antioxidant gene expression (Civelek et al., 2017; Coleman et al., 2018; Merry & Ristow, 2016), to be significantly impacted in muscle from PolG^+/mut^ versus control mice (Figure 6g, *p* < .05).

In total, findings from proteomic analyses of muscle mitochondria from 12‐month‐old male PolG^+/mut^ mice revealed that mtDNA point mutations drive changes in the mitochondrial proteome and relevant upstream transcription factors. In our view, these alterations are likely compensatory for the preservation of metabolic homeostasis.

## 3 DISCUSSION

The overall goal of the present research was to determine whether high‐level mtDNA point mutations disrupt metabolic health. Our findings indicate that increased mtDNA point mutations fail to induce insulin resistance, adiposity, or broad changes in markers of mitochondrial function related to the genome, proteome, and metabolome of postabsorptive 6‐month‐old PolG^+/mut^ mice. Moreover, mtDNA point mutations also failed to impair the metabolic response to nutritional challenge imposed by 24‐hr starvation or HFD feeding. Although aging blunted body weight gain between 14 and 40 weeks in PolG^+/mut^ versus control mice, no gross reduction in tissue weights or deterioration of metabolic health as a consequence of increased mtDNA point mutations was observed. However, aspects of the skeletal muscle mitochondrial proteome as well as transcription factors known to regulate mitochondrial biogenesis and metabolic function were differentially expressed in PolG^+/mut^ versus control at 12 months of age. These findings suggest that despite elevated point mutations, cells adapt to overcome this molecular challenge even in the face of aging. Our findings are generally in line with Vermulst et al. showing that mtDNA point mutations in male PolG^mut/+^ mice fail to impact health span or lifespan (Vermulst et al., 2007, 2008).

Our findings in middle‐aged mice raise an important question regarding compensatory mtDNA–nuclear(n)DNA signaling that occurs to preserve metabolic health in the context of DNA instability. Since nearly all mitochondrial proteins are nuclear‐encoded (~4,900 in total), heightened mitochondrial protein abundance suggests nuclear compensation to overcome the stress imposed by a high mitochondrial DNA point mutation frequency. Increased expression of mitochondrial ribosomes to enhance protein translation as well as increased expression of Sod1, Sod2, Prdx2, Txnrd2, Ucp3, Park7, Glxr2, Romo1, Cat, and Bnip3 in muscle of 12‐month‐old mice versus aged‐matched controls is indicative of oxidative stress in muscle of the PolG^+/mut^ mice. Observations in human subjects show that defects in mitochondrial protein synthesis drive varying degrees of electron transport chain impairment and subsequent oxidative stress (Jacobs, 2003; Rotig, 2011). We speculate that the alteration in the mitochondrial proteome coordinated by the nuclear and mitochondrial genomes might be an initial feedback mechanism to preserve mitochondrial function and that to induce decrements in insulin action and muscle performance in the PolG^+/mut^ mouse would require a metabolic stress more severe than dietary intervention.

Normal chow‐fed postabsorptive mice showed increased muscle mtDNA copy number at ages 6 and 12 months which is suggestive of increased mtDNA replication or reduced mitophagic flux, or both. These data are in contrast to the mtDNA depletion phenotype of the PolG^mut/mut^ mouse and polg‐1(srh1) *C. elegans* in which copy number was reduced > 50% (Haroon et al., 2018; Trifunovic et al., 2004; Vermulst et al., 2008). Moreover, in contrast to PolG^mut/mut^, PolG^+/mut^ mice showed no change in mtDNA replication capacity (He, Shumate, White, Molineux, & Yin, 2013); therefore, increased mitochondrial DNA replication is a possible mechanism of compensation to elevate the abundance of healthy DNA in muscle of PolG^+/mut^ mice. Although gene and protein markers of autophagy were identical between the groups, a limitation of this work is that a more sensitive assessment of in vivo mtDNA turnover was not performed; thus, the mechanisms contributing to the copy number elevation of the mt genome remain unclear.

Because mtDNA mutations are thought to accumulate sporadically with aging, we cannot exclude that mutations did not reach a heteroplasmic threshold to elicit the proteomic response in the 12‐month‐old PolG^+/mut^ mice. In vitro and in vivo data indicate that when specific thresholds of heteroplasmy are achieved, unique phenotypes are observed (Kopinski et al., 2019; Picard et al., 2014; Williams et al., 2010). Recent work by Kopinski et al. shows that mtDNA heteroplasmy in cybrid cell lines causes marked changes in the production of mitochondrial citric acid cycle intermediates that are substrates for epigenetic modification (Kopinski et al., 2019). Another limitation of this research is that we did not perform metabolomics in the young versus aged muscle to understand how mtDNA instability affects specific regulatory metabolites. However, since our data predict robust activation of a variety of transcription factors including the histone demethylase KDM5A which is responsive to α‐ketoglutarate, and since hierarchical clustering uncovered a distinctive mitochondrial proteome, we postulate that a unique metabolomic signature would be revealed in muscle from PolG^+/mut^ mice.

In human subjects, the overall prevalence of mtDNA mutations is ~ 1:5,000 (Lightowlers, Taylor, & Turnbull, 2015). Interestingly, disease outcomes associated with mitochondrial DNA mutations are highly variable (Wallace, 2018). Not only is the degree of mitochondrial mutation heteroplasmy a consideration, but emerging research shows that specific mtDNA variants can modify the nuclear genome expressivity, and thus, the coupling of mtDNA and nDNA defects presumably gives rise to varying outcomes (McManus et al., 2019). Case in point, the presence of specific sub‐pathogenic mtDNA variants on the nuclear DNA *Ant^‐/‐^* background was shown to cause wildly different effects on lifespan (McManus et al., 2019), and in one extreme instance reduced the median lifespan by 35%. A limitation of the present study is that the research was performed on one genetic background strain, C57Bl/6J. In addition, mice were only aged to 12 months, which is considered middle age. It would be of interest to study the effects of high mtDNA point mutations on different inbred strains of mice at a later time point (>24 months) to identify nuclear genome variants that accentuate pathogenic phenotypes of mtDNA instability.

Disorders related to POLG functionality are a major cause of mitochondrial disease (Copeland, 2008, 2012) delivering varying phenotypes in humans. It is reported that there are 176 unique point mutations specifically in the *POLG1* gene that encodes the catalytic subunit of the POLG enzyme (Nurminen, Farnum, & Kaguni, 2017). Considering the wide range of phenotypic outcomes observed in humans with POLG1 mutations near amino acid 257 (mutator mouse PolG D257A), several groups have suggested multiple roles for the POLG protein beyond mtDNA replication and repair (Copeland, 2012, 2014; Stumpf, Saneto, & Copeland, 2013). These human studies along with findings linking mitochondrial genome instability with numerous chronic diseases that plague society (Czajka et al., 2015) support a strong justification for follow‐up studies in rodents and relevant cell systems to determine the full scope of POLG action. A better understanding of the regulatory mechanisms of this complex enzyme, including the specific actions of the proofreading domain versus the polymerase active site, and the contributions of these sites to metabolism and cellular aging will likely exert a strong impact on the field and advance clinical care of patients suffering from mitochondrial‐related disorders.

## 4 EXPERIMENTAL PROCEDURES

### 4.1 Ethical approval

This study was approved by the University of California, Los Angeles Institutional Animal Care and Use Committee. All animal care, maintenance, surgeries, and euthanasia were conducted in accordance with this Institutional Animal Care and Use Committee and the National Institutes of Health.

### 4.2 Animal models and diets

Jackson (Bar Harbor, ME, USA) 017341 (background: B6.129S7; genotype: PolG^+/mut^) laboratory mice were purchased and crossed to generate control (Control or PolG^+/+^) and polymerase gamma heterozygous (PolG^+/mut^) littermate mice. Female PolG^+/+^ (WT) were bred with male PolG^+/mut^ for all studies as previously described (Ross et al., 2013). Mice were group‐housed two to four per cage and fed chow diet ad libitum (8604, Teklad; calories: 25% protein, 14% fat, 54% carbohydrate) unless otherwise indicated. Male mice were used for all studies to avoid sex as a confounding variable. Mice were fasted for 6 or 24 hr prior to euthanasia as indicated. High‐fat fed mice had ad libitum access to high‐fat chow that consisted of the following: 45% fat, 20% protein, and 35% carbohydrates (D12451; Research Diets, Inc., New Brunswick, NJ, USA). The 24‐hr starvation protocol followed standard laboratory practices for fasting mice (Jensen, Kiersgaard, Sørensen, & Mikkelsen, 2013).

### 4.3 Grip strength, maximal running speed, dynamic hanging, and run to exhaustion tests

Mouse genotypes were blinded to the experimenter for all tests. Grip strength was assessed using the GT3 Grip Strength Meter (Bioseb, Pinellas Park, FL, USA). Each mouse performed five trials, and the highest three trials were averaged. Maximal running speed was assessed as described previously (Lerman et al., 2002). Briefly, mice were acclimated to the running treadmill on two separate occasions prior to the maximal running speed test. On testing day, mice performed a 5‐min warm‐up at 5–10 m/min. Treadmill speed was increased by 3 m/min until mice were unable to maintain the speed for 10 consecutive seconds with gentle encouragement. Mice were given three attempts at each speed and approximately 60 s of rest after each increase in treadmill speed. Dynamic hanging as assessed by a latency to fall test, an index of grip strength and muscle endurance, was performed as previously described (Mandillo et al., 2014). Mice were acclimated to the wire grid on two separate occasions prior to testing. Mice performed three trials, and the data were averaged and reported as a mean ± *SEM*. Mice were given five min of rest between each trial. Run to exhaustion was performed as described previously (Fan et al., 2017). Briefly, mice were acclimated to treadmill running on two separate occasions prior to the test in which a 5‐min warm‐up was followed by an incremental increase in treadmill speed (3m/min) starting from 10 m/min (fixed 5° incline). Running speed intervals were 3 min in length, and the test was terminated when mice could no longer perform the running exercise (indicated by > 10 s of inactivity on the resting platform with gentle encouragement).

### 4.4 Glucose and insulin tolerance testing

Glucose and insulin tolerance tests (GTT or ITT) were performed following a 6‐hr fast as previously described (Ribas et al., 2016). Briefly, the GTT consisted of an intraperitoneal dextrose (1 g/kg) injection and glucose was assessed at 15‐min intervals over 120‐min testing period. The ITT consisted of an intraperitoneal insulin injection (1.0 U/kg). Blood samples were drawn, and glucose was measured at 0, 15, 30, 45, 60, 90, and 120 min post‐injection.

### 4.5 Immunoblot analysis

Whole quadriceps muscles from both legs were frozen in liquid nitrogen while being pulverized together into a powder. A homogenous sample of pulverized muscle was used for immunoblotting (Drew et al., 2014). Proteins from each individual whole‐cell homogenate were normalized (expressed relative to the pixel densitometry) to glyceraldehyde 3‐phosphate dehydrogenase (GAPDH, AM4300; Ambion, Foster City, CA, USA). Phosphorylation‐specific proteins were normalized (expressed relative to pixel densitometry) to the same unphosphorylated protein (i.e., phosphorylated Drp1 at Ser 616 was expressed relative to the pixel densitometry of Drp1 for each individual sample). In many cases, membranes were cut so that multiple proteins could be probed with different antibodies simultaneously. This allows for conservation of sample and reagents. Phosphoprotein blots were stripped and reprobed with antibody against the protein of interest. GAPDH protein or the mitochondrial‐specific HSP60 were assessed for every membrane to ensure equal loading of all lysates. See Table S1 for a list of the primary antibodies used.

### 4.6 DNA and RNA extraction, cDNA synthesis, and quantitative RT–PCR

DNA and RNA were extracted from a portion of pulverized frozen quadriceps muscle homogenate using DNeasy/RNeasy Isolation Kits (Qiagen, Germantown, MD, USA) as described by the manufacturer. Isolated DNA and RNA were tested for concentration and purity using a NanoDrop Spectrophotometer (Thermo Scientific, Waltham, MA, USA). Isolated RNA was converted into cDNA, assessed for purity, and qPCR of the resulting cDNA levels was performed as previously described (Drew et al., 2014). All genes were normalized to the housekeeping gene Ppia or 18S. Mitochondrial DNA content was assessed as a ratio of mitochondrial DNA (mtCO2) to nuclear DNA (18S). Primers used for qPCR can be found in Table S2.

### 4.7 Plasma analysis

Briefly, immediately following euthanasia whole blood was removed via 27‐gauge needle from the abdominal aorta, centrifuged at 2,000 *g* for 2 min in EDTA‐coated tubes. Assessment of plasma lactate was determined using the Eton Bioscience (San Diego, CA, USA) l‐Lactate Assay Kit I Protocol Version 7. Plasma iron level was determined using the Iron‐SL Assay (Sekisui Diagnostics, Lexington, MA, USA) following the manufacturer's protocol. Assessment of plasma triglyceride was determined using the L‐Type TG M Assay (Wako Diagnostics, Mountain View, CA, USA). Assessment of plasma glucose was determined using HemoCue Glucose 201 Systems glucometer.

### 4.8 Muscle or liver glycogen

Glycogen was assessed in excised quadriceps muscle and liver using the following adapted protocol (Dubois, Gilles, Hamilton, Rebers, & Smith, 1951). Briefly, ~100 mg of muscle or liver was weighed and cut into small pieces. 1 ml of 30% KOH was added and boiled for 25 min. The tubes were cooled to room temperature after which 2 ml of 95% ethanol was added. The tubes were then incubated for 30 min on ice, centrifuged at 550 *g* for 30 min at 4°C, and the resulting supernatant was removed and discarded. The pellets were dissolved in 1 ml of Mili‐Q H_2_O after which 1 ml of 5% phenol was added. The samples were further diluted 2:3 in Mili‐Q H_2_O and 5 ml of 96%–98% H_2_SO_4_. Following incubation on ice for 30 min, the optical density was measured at 490 nm. Concentrations were determined using an eight‐point glycogen standard curve and normalized to mg of tissue weight assayed.

### 4.9 Tissue histology

Tibialis anterior muscles were sectioned and stained for cytochrome c oxidase (COX) as previously described (Wanagat, Cao, Pathare, & Aiken, 2001).

### 4.10 Mitochondrial complex 1 activity assay

Mitochondria were isolated from gastrocnemius muscle as described previously (Philp et al., 2011). Mitochondrial complex 1 activity assay was performed following the protocol outlined by Philp *et al*. Briefly, 30 µl of isolated mitochondria was added to 0.05 M Kpi buffer, 0.2 mM NADH, 0.3 mM KCN, 20 µM antimycin A, and 0.05 M BSA. The above solution was preheated to 30°C after which 5 mM ubiquinone‐1 was added. Absorbance values were read at 340 nm every 15 s for 3 min after which 10 µM rotenone was added. Absorbance values were again read at 340 nm every 15 s for 3 min. Enzyme activity was determined as the delta of ubiquinone and rotenone absorbance/min/µg protein and normalized to average control values.

### 4.11 Mitochondrial DNA mutation assay

Mitochondrial DNA deletion mutations were measured in quadriceps muscles as previously described (Herbst et al., 2017). Briefly, mtDNA deletions were directly detected via the highly sensitive digital PCR method following an adapted established PCR protocol that limits the extension time allowing for the selective amplification of mtDNA deletions. This sensitivity is sufficient to quantitate the abundance of mtDNA deletions in young muscle homogenates down to a level of 0.03%. Mitochondrial DNA point mutation frequency was quantitated in mouse quadriceps muscles as previously described (Valente et al., 2016) using quantitative real‐time PCR (Vermulst et al., 2007). Briefly, mtDNA point mutations were detected using an adapted random mutation capture assay which includes TaqI restriction endonuclease digest allowing for cleavage of nonmutated mtDNA, while mutated mtDNA is resistant to cleavage. A control region resistant to TaqI cleavage within mtDNA is used as a reference.

### 4.12 Mitochondrial proteomic analysis

Mitochondria were isolated from gastrocnemius muscle. Muscles were Dounce‐homogenized, and then, mitochondria were isolated using the Mitochondria Isolation Kit for tissue (Thermo Scientific). Isolated mitochondria went through a Percoll density gradient for added purification (Graham, 2001). Isolated mitochondria were then lysed via buffer (0.5% sodium deoxycholate, 12 mM sodium lauroyl sarcosine, 50 mM triethyl ammonium bicarbonate (TEAB)) with bath sonication. Samples were treated with Tris (2‐carboxyethyl) phosphine, chloroacetamide, and incubated overnight with sequencing grade modified trypsin (Promega, Madison, WI, USA) following addition of equal volume ethyl acetate/trifluoroacetic acid. Supernatants were discarded after centrifugation, and resulting phase was desalted on a C18‐silica disk (3M, Maplewood, MN, USA) according to Rappsilber's protocol (Rappsilber, Mann, & Ishihama, 2007). The conllected eluent was chemically modified using a TMT10plex Isobaric Label Reagent Set (Thermo Fisher Scientific) as per the manufacturer's protocol, and an aliquot was taken for measurement of total peptide concentration (Pierce Quantitative Colorimetric Peptide, Thermo Fisher Scientific). The samples were then pooled according to protein content and fractionated via high pH reversed‐phase chromatography using a 1,260 Infinity LC System (Agilent Technologies, Santa Clara, CA, USA) and a ZORBAX 300Extend‐C18 column (Agilent Technologies, 0.3 × 150 mm, 3.5 μm). Twelve fractions were injected onto a reverse‐phase nanopore HPLC column (AcuTech Scientific) using an Eksigent NanoLC‐2D system (Sciex, Framingham, MA, USA). The effluent from the column was directed to a nanospray ionization source connected to a hybrid quadrupole‐Orbitrap mass spectrometer (Q Exactive Plus, Thermo Fisher Scientific) acquiring mass spectra in a data‐dependent mode alternating between a full scan (*m/z* 350–1700, automated gain control (AGC) target 3 × 10^6^, 50‐ms maximum injection time, FWHM resolution 70,000 at *m*/*z* 200) and up to 10 MS/MS scans (quadrupole isolation of charge states ≥ 2, isolation width 1.2 Th) with previously optimized fragmentation conditions (normalized collision energy of 32, dynamic exclusion of 30 s, AGC target 1 × 10^5^, 100‐ms maximum injection time, FWHM resolution 35,000 at *m/z* 200). The raw data were analyzed in Proteome Discoverer 2.2, providing measurements of relative abundance of identified peptides. Downstream analysis was performed using DAVID Bioinformatics Resources and Ingenuity Pathway Analysis.

### 4.13 Muscle respiration

Frozen skeletal muscle tissues were thawed on ice and homogenized in MAS (70 mM sucrose, 220 mM mannitol, 5 mM KH_2_PO_4_, 5 mM MgCl_2_, 1 mM EGTA, 2 mM HEPES, pH 7.4). The samples were mechanically homogenized with 60 strokes in a Teflon‐glass Dounce homogenizer. All homogenates were centrifuged at 1,000 *g* for 10 min at 4°C, and then, the supernatant was collected. Protein concentration was determined by BCA. Homogenates were loaded into Seahorse XF96 microplate in 20 μL of MAS at 6 µg/well. The loaded plate was centrifuged at 2,400 *g* for 10 min at 4°C (no brake), and an additional 130 μL of MAS supplemented with 100µg/ml cytochrome c was added to each well. Substrate injection was as follows: Port A: NADH (1mM) or succinate + rotenone (5 mM + 2 μM); Port B: rotenone + antimycin A (2 + 2 μM); Port C: N,N,N′,N′‐tetramethyl‐p‐phenylenediamine (TMPD) + ascorbic acid (0.5 + 1 mM); and Port D: azide (50 mM). These conditions allow for the determination of the maximal respiratory capacity of mitochondria through complex I, complex II, and complex IV.

### 4.14 Liver lipid measurements

Liver lipids were measured, quantified, and analyzed as previously described (Chella Krishnan et al., 2018).

### 4.15 Statistical analysis

Values are presented as mean ± *SEM* and expressed relative to the average value obtained for each experimental Control group unless otherwise stated. Group differences were assessed by Student's *t* test with statistical significance established a priori at *p* < .05 (GraphPad Prism, San Diego, CA, USA).

## CONFLICTS OF INTEREST

The authors declare no competing financial interests.

## AUTHOR CONTRIBUTIONS

Timothy M. Moore, Zhenqi Zhou, Alexander R. Strumwasser, Whitaker Cohn, Julian P. Whitelegge, Amanda J. Lin, Kevin Cory, Kate Whitney, Theodore Ho, Timothy Ho, Joseph L. Lee, Daniel H. Rucker, Kevin Widjaja, Aaron D. Abrishami, Sarada Charugundla, and Linsey Stiles performed the experimental work. Timothy M. Moore was involved in data analysis and interpretation. Timothy M. Moore and Andrea L. Hevener designed the study. Timothy M. Moore, Lorraine P. Turcotte, Jonathan Wanagat, and Andrea L. Hevener wrote the manuscript.

## Supporting information

Figure S1‐S5
