# Supplementary material for: Age‐induced mitochondrial DNA point mutations are inadequate to alter metabolic homeostasis in response to nutrient challenge
Source: Aging Cell. 2020 Oct 13;19(11):e13166. doi: 10.1111/acel.13166 (PMC7681042; doi:10.1111/acel.13166)
Supplement: Supplementary file 1 — Figure S1‐S5 [file ACEL-19-e13166-s001.pdf]

Supplemental Table 1. Primary Antibodies Used

Protein Name	Protein Abbreviation	Cat. #	Company
4E Binding Protein 1	4EBP1	9644	Cell Signaling
Acetyl CoA Carboxylase	ACC	3662	Cell Signaling
Protein Kinase B	Akt	9272	Cell Signaling
AMPK Alpha	AMPKa	2532	Cell Signaling
Autophagy Related 10	ATG10	A9356	Sigma-Aldrich
Autophagy Related 3	ATG3	3415	Cell Signaling
Autophagy Related 7	ATG7	2631	Cell Signaling
Adipose Triglyceride Lipase	ATGL	2138	Cell Signaling
Beclin-1	Beclin1	3495	Cell Signaling
Cluster Determinant 36	CD36	D-2712	Novus Biologicals
Mitochondrial OxPhos Complexes	CI - CV	B0315	MitoSciences
Diacylglycerol O-Acyltransferase 1	DGAT1	NB100-57086	Novus Biologicals
Parkinson Disease 7	DJ1	5933	Cell Signaling
Dynamin-related protein 1 Drp1	Drp1	8570	Cell Signaling
Mitochondrial Fission Protein 1	Fis1	GTX111010	GeneTex
Forkhead TF 1	Foxo1	2880	Cell Signaling
Glucose Transporter 4	GLUT4	G4048	Sigma-Aldrich
Hexokinase II	HKII	HXK23-A	Alpha Diagnostic
Heat Shock Protein 60	HSP60	12165	Cell Signaling
Insulin Receptor Substrate 1	IRS1	3407	Cell Signaling
MAP 1A/1B Light Chain 3B	LC3B	2775	Cell Signaling
Mitochondrial fission factor	MFF	ab81127	Abcam
Mitofusin 1	MFN1	75-162	NeuroMab
Mitofusin 2	MFN2	ab56889	Abcam
Mitochondrial Dynamics Protein of 51 kDa	MiD51	PA5-25483	Thermo Scientific
Optic atrophy 1	Opa1	612606	BD Biosciences
phosphorylated 4E Binding Protein 1 Thr 37/46	p-4EBP1 Thr 37/46	2855	Cell Signaling
phosphorylated Acetyl CoA Carboxylase Ser 79	p-ACC Ser 79	11818	Cell Signaling
phosphorylated Akt Ser 473	p-Akt Ser 473	9271	Cell Signaling
phosphorylated AMPK Alpha Thr 172	p-AMPKa Thr 172	2535	Cell Signaling
Parkin	Parkin	2132	Cell Signaling
phosphorylated Drp1 Ser 616	p-Drp1 Ser 616	4494	Cell Signaling
phosphorylated Drp1 Ser 637	p-Drp1 Ser 637	6319	Cell Signaling
phosphorylated Forkead TF 1 Ser 256	p-Foxo1 Ser 256	9461	Cell Signaling
PPAR Gamma Coactivator 1 Alpha	PGC1a	AB3242	Millipore
PTEN Induced Putative Kinase 1	Pink1	10006283	Cayman Chemical
Perilipin 2	Plin2	03-610102	ARP
Perilipin 5	Plin5	NB110-60511	Novus Biologicals
DNA Polymerase Gamma	PolG	SC-48815	Santa Cruz
phosphorylated Parkin Ser 65	p-Parkin Ser 65	ab154995	Abcam
phosphorylated Autophagy Related 1 Ser 467	p-Ulk1 Ser 467	4634	Cell Signaling
Sequestosome 1	SQSTM1 or p62	5114	Cell Signaling
Transcription factor A, Mitochondrial	TFAM	ab131607	Abcam
Autophagy Related 1	Ulk1	A7481	Sigma-Aldrich

Supplemental Table 2. qPCR Primers

GeneName	Forward Primer	Reverse Primer
ABCB10	CTGTCAGCTGCAGTTGGGTT	TGAGGTAGACACGAATGCCG
ACACA	CTGAAGCAGATCCGCAGCTT	GGTGAGATGTGCTGGGTCATG
ACACB	CATACACAGAGCTGGTGTTGGACT	CACCATGCCCACCTCGTTAC
ACADL	GTAGCTTATGAATGTGTGCAACTC	GTCTTGCGATCAGCTCTTTCATTA
ACADM	GCAGCCAATGATGTGTGCTTAC	CACCCTTCTTCTCTGCTTTGGT
ACOX1	GCCCAACTGTGACTTCCAT	GGCATGTAACCCGTAGCACT
ACSL1	ACCATCAGTGGTACCCGCTA	CTTCCAACCAACACCCTCAT
ATG12	CCACAGCCCATTTCTTTGTT	GAAACAGCCACCCCAGAG
ATG3	ATTCTTCCCCTGTAGCCCAT	GAAGTGGCCGAGTACCTGAC
ATG5	GATCTCCAAGTGTGTGCAGC	TCCAGAAGAAAATGGATTTCG
ATG7	GCCAGGTACTCCTGAGCTGT	ACTTGACCGGTCTTACCCTG
Beclin1	CCCCGATCAGAGTGAAGCTA	AGGAGAGACCCAGGAGGAAG
C/EBPa	CCCCCACTCAGCTTACAACAGG	CACCCCACAAAGCCCAGAAAC
CD36	TCCAGCCAATGCCTTTGC	TGGAGATTACTTTTTCAGTGCAGAA
CO1	ACTATACTACTAACAGACCG	GGTTCTTTTTTTCCGGAGTA
CO2	CTACAAGACGCCACAT	GAGAGGGGAGAGCAAT
CO3	GCAGGATTCTTCTGAGCGTTCT	GTCAGCAGCCTCCTAGATCATGT
CPT1B	CTCCTGGAAGAAACGCCTTATT	CACCTTGCAGTAGTTGGAACC
DGAT1	AGGATCAGCATCACCACACA	GACGGCTACTGGGATCTGA
DGAT2	CTGTTGAGCCAGGTGACAGA	CGCAGCGAAAACAAGAATAA
DNM1L	CGTGGACTAGCTGCAGAATG	TGCCTCAGATCGTCGTAGTG
FABP4	TTCGATGAAATCACCGCAGA	GGTCGACTTTCCATCCCACTT
FASN	TGCTCCCAGCTGCAGGC	GCCCGGTAGCTCTGGGTGTA
FGF21	CCTCTAGGTTTCTTTGCCAACA	AAGCTGCAGGCCTCAGGAT
Gfm2	ACCGTCCAACACCCTCAAG	AAGAACGAGAAAGGGGCATT
GPAM	TCATCGAGCCTCCGTCTTAT	CACCTTCATCCTCTTTTGCC
GPX3	GATGGTGAGGGCTCCATACT	CATCCTGCCTTCTGTCCCT
HSPA9	GGGCAAACAAGCAAAGGTCC	TTGCCGTTTTGCTGGCATAC
HSPD1	CTCACTCGCCGCAGACG	TGGGTAGTCGAAGCATTTCTGC
HSPE1	CCTTTTCACGTGTCCCAGCC	TTGTCCAGCCATGACTCTCG
IFNG	AGCAACAGCAAGGCGAAAA	CTGGACCTGTGGGTTGTTGA
IL10	GGTTGCCAAGCCTTATCGGA	ACCTGCTCCACTGCCTTGCT
IL6	AGTCCGGAGAGGAGACTTCA	TTGCCATTGCACAACTCTTT
JMJD3	GTCAGCCTCATAGCAGGACC	CGCCTCAGTAACAGCCAGAT
LPIN1A	TTTTTGCATACAAAGGCAGC	GGGGTTCAGTCCCTTGTAGAG
LPIN1B	ACAGTGCCTGACTGGGAAAG	CAAGAGAGAGAGGTGGCTCG
LPL	AGGACCCCTGAAGACAC	GGCACCCAACTCTCATA
MAPLC3B	AATCACTGGGATCTTGGTGG	AGTCAGATCGTCTGGCTCG
Mgme1	TGTTAGGTTCTCCTGGGGTG	TTTGGAGAGGTGGAAAGAGC
ND1	GTTGGTCCATACGGCATTTT	TGGGTGTGGTATTGGTAGGG
ND2	CGCCCCATTCCACTTCTGATT	TTAAGTCCTCCTCATGCCCCT
ND4	GCCTGATTACTGCCACTAATA	GGTTCCCTCATCGGGTAATAA
ND4L	ACTATCACTTCTAGGGACACT	TTGGACGTAATCTGTTCCGT
ND5	AACCACACCTAGCATTCCTAC	CAGGCGTTGGTGTTGCAGGTA
ND6	ACAACTATATATTGCCGCTAC	GATATACGACTGCTATAGCTA
NRF1	GAACTGCCAACCACAGTCAC	CGTCTGGATGGTCATTTCAC
Park2	ATCGACCTCCACTGGGAAG	GCGTAGGTCCTTCTCGACC
Peo1	GCCCAGTCACCAGTTTCCTA	ACTCTGGTCATTCACCCTCG
Perilipin1	ACAGCAGAATATGCCGCCAA	GGCTGACTCCTTGTCTGGTG
PGC1α	TGAGGACCGCTAGCAAGTTT	TGAAGTGGTGTAGCGACCAA
Pink1	GGATGTCGTCCTGAAGGGAG	GCTTCGCTGGAGGAACCTG
PolG	TAGCTGGCTGGTCCAAGAGT	CGACGTGGAGGTCTGCTT
PolGII	CCGTTTTCCAGCGTAGTCTC	TTCTGTGTGGCCTGGCTATT

Polrmt	CTCATCTCAGGTGTGCCCTC	TCTGCAGCTCAAGAAGGAGC
PPARg	GCCCTTTGGTGACTTTATGG	CAGCAGGTTGTCTTGGATGT
SATB1	AAGTCGCCTTCAGGTCTGC	TCACCTGCCAGAACACTTCA
SDHA	TACTACAGCCCCAAGTCT	TGGACCCATCTTCTATGC
SOD2	AACTCAGGTCGCTCTTCAGC	GCTTGATAGCCTCCAGCAAC
SQSTM1	TTTCTGGGGTAGTGGGTGTC	CTGAAGAATGTGGGGGAGAG
TFAM	AGCTTGTAAATGAGGCTTGGA	AGATGTCTCCGGATCGTTTC
TNFA	CCAGACCCTCACACTCAGATC	CACTTGGTGGTTTGCTACGAC
TXN1	CCCTTCTTCCATTCCCTCTGT	CGGCATGCATTTGACTTCAC
TXN2	CTAGCTGGTCCTCGTCCTTG	TCACACAGACCTTGCCATTG
TXNIP	CAAGGGTCTCAGCAGTGCAA	TTTGTTTCCAGGCCTCATGAT
TXNRD1	CCAAGACACTCCCCTCCTCAT	GGCAGCTCAGATTGCGTATTTT
Ulk1	TAGTCAGCCAGGTCTCCACC	CTGCTGGGAAAGGAAATCAA

Accession	Fold Change	-LOG(P-Value)
E9QPQ8	0.18506	2.48728
Q9CPR5	0.16548	2.16821
Q9D7J4	0.25887	2.16556
Q99N15	0.20529	2.05299
Q9CQE3	0.50790	1.94785
Q9CY73	0.32883	1.92774
Q9Z2G9	0.37473	1.86655
Q9CXJ4	-0.13294	1.85724
Q9Z2Y8	-0.15653	1.85467
D3Z1Z1	0.24361	1.80393
P80313	-0.23930	1.79223
Q9CZR8	0.41196	1.78752
Q9CPW9	0.18007	1.75630
Q3URE1	-0.13049	1.72437
E0CYB9	0.24540	1.71844
P24270	-0.08520	1.71045
Q9D1I6	0.23819	1.68801
P09528	-0.24209	1.66934
Q8CG76	0.19202	1.66442
E9QPD7	0.15750	1.65874
Q3UUI3	0.47182	1.65191
Q8C6I2	0.14986	1.64600
Q8BK72	0.21413	1.64414
P09103	0.20189	1.63428
Q9D0E1	0.34844	1.61827
Q6XPS7	0.26193	1.60933
Q80Y14	0.36742	1.60656
P20108	0.21288	1.60165
Q9CWD8	0.22767	1.56947
Q9CPU4	-0.12329	1.51648
Q9D3P8	0.28961	1.51310
Q9DCM0	0.11149	1.50736
Q7TNG8	0.46391	1.47939
Q9D2R6	0.30127	1.47275
Q9CQY6	0.13377	1.46233
Q9CR61	0.12450	1.45923
Q99PT1	-0.23429	1.44550
P97478	0.13017	1.43696
Q8VCW8	0.19877	1.43005
F8VQJ3	-0.33389	1.41647
Q8BHE8	0.21631	1.41408
Q544X6	0.16638	1.41333
Q8JZN7	0.13538	1.41067
Q6NVE9	0.09621	1.40871
Q8BGT5	0.27044	1.40429
G5E8T9	0.25785	1.39193
Q3TQB2-2	0.38355	1.37725
Q91VC9	0.46816	1.36900
Q3UMR5	0.23214	1.36037
E9QMK9	0.17606	1.35387
Q8BGH2	0.13675	1.34951
Q8R4N0	0.17155	1.34345

Accession	Fold Change	-LOG(P-Value)
Q9CWT6	0.40616	1.33323
Q9D8P4	-0.13572	1.32700
P62075	-0.15464	1.32603
G5E814	0.32072	1.31954
Q8BH59	0.24697	1.31142
Q8CC21	0.39521	1.30290
P59041	0.29779	1.29888
Q78YY6	-0.20581	1.29708
Q9CZL5	-0.14280	1.29439
Q9D880	0.20163	1.29340
Q920A7	0.31566	1.26986
Q9CPX7	0.38493	1.24377
Q9JHW2	0.09817	1.21883
Q8BKZ9	0.22195	1.21337
Q60930	0.28102	1.20943
D3Z285	0.16263	1.20695
Q9CR59	0.13083	1.20411
Q64521	0.18719	1.15170
Q99J47	0.19974	1.13476
Q91VA7	0.15146	1.13475
P58281-2	0.17344	1.13052
Q8BMD8	0.43132	1.12462
Q91VA6	0.17960	1.12328
Q9CQ85	0.24646	1.11868
Q9Z2I9	0.26546	1.11798
Q9CZ83-2	0.24517	1.11594
Q3V384	0.16008	1.11356
Q60932	0.23892	1.11014
E9PUD2	-0.12769	1.10387
Q8BMF4	0.20675	1.10181
Q9DB73	0.27112	1.09465
P97742	0.25378	1.08295
Q78PY7	-0.28078	1.07638
Q9D051	0.19539	1.07538
Q9EQ20	0.10061	1.07210
Q9D6Y7	-0.08354	1.06998
P97807	0.14408	1.05612
Q8R404	0.17865	1.05215
Q9CWB7	0.23414	1.05185
O88967	0.10748	1.05108
Q8R0F8	0.16475	1.04918
Q9CQ91	0.19565	1.04673
P63038	0.19276	1.04256
Q99LC5	0.11709	1.03610
Q91YT0	0.17108	1.02935
Q3UHB1	0.20631	1.02361
Q8R2Q4	0.11775	1.01974
Q99JB2	0.12314	1.01894
Q9D3D9	0.18553	1.01677
Q99N87	0.12314	1.01361
Q9CQN1	0.09512	1.00957
Q9R112	0.14503	1.00900

Accession	Fold Change	-LOG(P-Value)	Accession	Fold Change	-LOG(P-Value)
P56480	0.16732	1.00850	O35972	-0.13919	0.85542
Q91YP0	0.09996	1.00221	Q9CR21	0.14087	0.85268
Q60931	0.23159	0.99955	O35129	0.17271	0.84978
Q9EQI8	0.13241	0.99891	Q921S7	0.07576	0.84957
Q9D2G2	0.14457	0.99298	Q9D1P0	0.07211	0.84499
Q80XL6	-0.17203	0.99025	O08528	0.10172	0.84465
Q9CQX2	-0.11321	0.98226	Q9QZ23	0.18410	0.84410
G5E8U5	0.35239	0.98201	Q99KI0	0.12811	0.84335
Q99KE1	0.16638	0.98079	B2RPU8	-0.14986	0.84277
Q9D1I5	0.07879	0.97909	Q9DBG1	0.17083	0.84267
Q9D273-2	0.20459	0.97777	Q9CWU6	0.18220	0.84245
Q8JZY4	-0.07191	0.97366	Q3UW66	0.13083	0.84065
Q9CPW2	-0.10498	0.96585	Q4VAE3	-0.14983	0.83828
Q8BH55	-0.14039	0.96139	Q8JZQ2	0.12383	0.83804
Q8R3J4	-0.45837	0.95974	Q9CQA3	0.14408	0.83425
P56135	0.22993	0.95468	Q8VDK1	0.40303	0.82507
Q791V5	0.22101	0.95057	P97450	0.13198	0.82202
Q91WS0	0.20870	0.95013	D3Z7X0	0.10284	0.82075
Q8BQU3	-0.19755	0.94963	Q922Q1	0.10748	0.81780
Q9CQN7	0.14454	0.94792	Q9Z2Q5	0.14500	0.81471
Q99KR7	0.15193	0.94688	Q5XJF6	-0.24834	0.80911
Q9CXV1	0.20919	0.94607	Q9DB15	0.11909	0.80725
Q8BFP9	0.12404	0.94324	Q9D6J6	0.13493	0.80708
O08749	0.16638	0.94204	P70404	0.08702	0.80598
Q8R127	0.24293	0.94201	Q03265	0.15378	0.80552
P35486	0.15239	0.93518	P40630	0.17533	0.80382
Q9DCW4	0.15610	0.93129	Q9CQL6	0.15657	0.80094
Q9D5T0	0.16822	0.93051	Q8C5Q4	0.09357	0.80043
B8JJ69	0.35738	0.92407	Q9CZ13	0.15171	0.80026
D3Z041	0.12585	0.92282	P56395	-0.09070	0.80008
Q9CR24	0.33178	0.92105	O35943	0.11149	0.79780
Q99NB1	0.22124	0.91400	Q8VE22	0.05760	0.79710
Q9CWE0	0.18150	0.90858	Q923X4	0.13837	0.79660
Q64433	0.19684	0.90218	Q925N1	-0.23322	0.79409
Q9CPY7	0.12926	0.90215	Q9D0S9	0.26934	0.79356
Q9CQB4	0.13083	0.90125	Q9WUM5	0.16873	0.79245
A2AJQ0	0.15728	0.90098	Q9D0G0	0.14246	0.78914
Q8R1I1	0.12993	0.89728	E9Q800	0.14546	0.78684
P03930	0.26165	0.89693	A2AKU9	0.12338	0.78328
Q9DCX2	0.16310	0.89683	Q8CGK3	0.10748	0.78275
Q3U2A8	0.17130	0.89626	Q8BMF3	0.19635	0.78145
P26443	0.09556	0.89388	Q8QZT1	0.14798	0.77283
G5E895	0.19662	0.88860	Q505D7	-0.15089	0.77110
Q8BFR5	0.11060	0.88572	Q8BJZ4	0.09843	0.76929
P00405	0.17796	0.88471	Q9DC70	-0.05428	0.76771
Q9DCC8	0.20022	0.88289	Q9JHS4	0.09514	0.76007
P99029	0.12471	0.87830	Q8K0Z7	0.14638	0.75849
Q9D404	0.13308	0.87719	Q80XN0	0.15189	0.75625
P51881	0.08464	0.87579	Q9DCT2	0.09357	0.75514
Q62425	0.16008	0.86758	Q9D6R2	0.11193	0.75183
Q9CQ69	0.24065	0.85993	A2AIL4	0.16638	0.74878
Q8JZN5	0.12495	0.85726	Q9D6U8	0.08988	0.74719

Accession	Fold Change	-LOG(P-Value)
G3X9F4	0.23892	0.74506
P67778	0.14316	0.74369
P56382	0.18103	0.74241
Q8BW75	0.13107	0.74232
P56392	0.08507	0.73849
Q9Z2I0	0.09975	0.73657
Q921H9	0.11685	0.73607
P08249	0.15100	0.73404
Q9CRB9	0.14273	0.73384
Q9CPQ3	0.17039	0.73120
P38647	0.13837	0.72857
Q9QUJ7	0.19732	0.72633
Q8CC88	-0.03401	0.72556
Q9CZU4	-0.12645	0.72195
P48962	0.11909	0.71953
Q9D0Q7	0.09885	0.71921
B1AXP6	0.18124	0.71732
A2AQ17	0.19517	0.71681
O09111	0.17391	0.70882
P09671	0.13038	0.70874
Q9D125	0.17770	0.70718
Q99M87	0.09820	0.70679
Q922D8	-0.12804	0.70643
Q9CQH3	0.13244	0.70162
Q9CQB5	0.12359	0.70155
Q07417	0.08876	0.69738
Q99MN9	0.07513	0.69527
Q61733	0.13423	0.69338
P16332	0.05825	0.69250
Q9CZW5	0.14525	0.69188
Q8R164	0.02902	0.68963
Q99LC3	0.13950	0.68693
Q99L13	0.07791	0.68668
Q9Z2I8	0.10328	0.68633
P05202	0.13017	0.68494
Q60759	0.13402	0.68075
Q8VCL2	0.05086	0.68002
Q9CQZ5	0.08030	0.67772
O35855	0.11796	0.67766
O70571	0.06825	0.67598
Q9ERS2	0.12540	0.67555
P52196	0.13289	0.67441
P56501	0.34380	0.67269
Q06185	0.14522	0.67058
Q9DCS9	0.08791	0.66930
P56391	0.16241	0.66850
B1AR13	0.10040	0.66741
Q8C3X4	-0.09596	0.66705
P29758	0.08420	0.66479
Q9CZD3	0.16191	0.66374
P38060	0.11172	0.66019
P99028	0.14090	0.66010

Accession	Fold Change	-LOG(P-Value)
Q9D6J5	0.16681	0.65785
Q9CQR4	0.07319	0.65408
Q8C3X2	-0.07228	0.65019
P97823	0.10571	0.64931
Q6PB66	0.10858	0.64905
P08074	0.09315	0.64328
Q8K2M0	0.10284	0.63987
Q8K0D5	0.10482	0.63968
Q8CBY0	0.24820	0.63817
Q9D8Y1	0.08139	0.63369
Q3U3J1	0.06719	0.63095
Q9WV96	-0.20032	0.62730
Q9CR62	0.15471	0.62690
Q8QZS1	0.10858	0.62414
Q8BX10-2	-0.09089	0.62241
Q9CR68	0.11843	0.62073
Q8K1Z0	0.10837	0.61779
Q9D7P6	-0.20503	0.61515
Q91YY4	-0.11017	0.61239
Q9DCS3	0.08094	0.60953
Q9JK42	0.06356	0.60706
Q8BMS1	0.10994	0.60458
Q99N93	0.09686	0.60228
Q9Z2Z6	0.10196	0.60212
Q9CWG8	-0.19215	0.60104
P50171-2	0.14844	0.59902
P00416	-0.20209	0.59726
Q811U4	0.05086	0.59715
Q9WTP6	0.16216	0.59685
Q9D0M3	0.13332	0.59572
Q61171	0.12902	0.59192
P14152	0.15471	0.58535
Q99LX0	0.08704	0.58197
Q9R0X4	0.07748	0.58131
Q9JLZ3	0.10084	0.58035
Q8BP40	0.08681	0.57858
Q60936	0.09952	0.57584
Q9JIK9	0.10704	0.57484
Q8BWM0	0.10947	0.57032
Q9EP89	0.15657	0.56943
Q9WV98	0.15678	0.56807
Q9CQ40	0.16804	0.56666
Q91WD5	0.08399	0.56395
Q924X2	0.07491	0.56248
Q9D6M3	0.23836	0.56109
Q9CQF4	0.02881	0.56045
P47934	0.10107	0.55852
B1ATI0	-0.22776	0.55733
Q9DCB8	-0.06520	0.55682
Q9CZP5	0.06186	0.55678
G5E902	0.11978	0.55513
Q3TC72	0.11240	0.55385

Accession	Fold Change	-LOG(P-Value)
Q9CWJ9	0.26883	0.55331
Q8BIJ6	0.08073	0.55318
A2AAJ9	-0.12769	0.55043
Q8BYL4	0.30840	0.55035
O35683	0.21506	0.54688
Q9JHI5	0.09994	0.54536
P47968	0.07598	0.54368
Q3U213	-0.10661	0.54209
Q924T2	0.15378	0.54198
Q9CQJ8	0.11282	0.54196
Q91WM2	0.06933	0.54061
Q8K411	0.06376	0.53991
O55028	0.10994	0.53855
Q9Z1J3	-0.05863	0.53593
Q5U458	0.10592	0.53566
Q9QZH6	0.09403	0.52863
Q9CQQ7	0.10438	0.52242
Q8VDT9	0.07987	0.52166
O35680	-0.16195	0.51057
Q9D009	-0.09340	0.50992
P45952	0.06591	0.50661
P17751	0.06441	0.49979
P03921	0.32365	0.49967
Q925I1	0.05402	0.49925
Q91ZA3	0.06740	0.49871
Q9CPZ8	0.12902	0.49769
Q9DC69	0.10727	0.49601
Q61425	0.09336	0.49526
A8Y5T6	-0.07136	0.49382
Q8BGC4	-0.07951	0.49133
Q80U63	0.09843	0.49019
Q9DBL1	0.07189	0.48742
Q8BMS4	0.07793	0.48244
Q9CZB0	0.05212	0.48182
Q80VL1	0.16263	0.47980
Q9D7B6	0.05846	0.47978
Q8K2Y7	-0.13103	0.47713
Q76LV0	0.07855	0.47503
P58064	-0.09869	0.47485
Q8BGY7	0.10393	0.46966
A2AMZ4	0.07209	0.46790
Q9CQB7	0.25654	0.46738
Q9D023	0.07512	0.46386
Q9CYF5	-0.30755	0.46062
Q9CQ75	0.15750	0.45937
Q99N94	-0.04764	0.45642
Q924D0	0.03666	0.45122
Q99M01	-0.14437	0.45085
G3X975	-0.13468	0.44979
P85094	0.07147	0.44792
O35143	0.16663	0.44785
Q5M8N4	0.06825	0.44636
Q9DB77	0.09929	0.44602
Q8K2B3	0.09161	0.44531
Q99M04	-0.07006	0.43786
Q8BH95	0.06313	0.43576
Q9CQ62	0.06719	0.43556
Q99KR3	-0.25004	0.43405
Q8BTX9	-0.11641	0.43287
Q9CZN7	0.10105	0.42504
Q921G7	0.07448	0.42224
G3X9M0	-0.08245	0.42003
Q9CPP6	0.06079	0.41620
Q9CPU2	0.15914	0.41529
Q14C51	0.04918	0.41485
Q9JJL8	0.08768	0.40970
Q91YM4	0.06485	0.40905
P00158	0.15889	0.40713
Q9D8S9	-0.08538	0.40600
Q8VCF0	0.24444	0.40472
O35857	0.08290	0.40470
P46656	0.10837	0.40362
Q99LP6	-0.02235	0.40004
Q8R2P8	-0.12648	0.39973
Q9DA03	0.12561	0.39548
Q99N96	0.07534	0.39376
Q8CHT0	0.09359	0.39228
Q9D8T7	0.09161	0.39141
Q9CXI0	0.03148	0.39053
P62073	-0.06539	0.38873
Q9D0L7	0.08660	0.38833
Q9CQ06	0.07146	0.38767
Q9CQU0	0.04730	0.38702
Q7TSQ8	0.04352	0.38655
Q8BGA9	0.09732	0.38618
Q9QYR9	0.05445	0.38562
Q91VD9	0.06484	0.38185
Q8K4Z3	0.05804	0.38107
P56394	-0.09250	0.37760
Q8R3Q6	-0.04458	0.37693
O08807	-0.11090	0.37588
P50544	0.07448	0.37306
P35385	-0.09777	0.37255
Q8K009	-0.13224	0.36709
Q9CR13	-0.10985	0.36630
D3Z7P3-2	0.08267	0.36586
Q8K3A0	0.11796	0.36035
Q3U125	0.05255	0.35943
Q8BVI4	0.11933	0.35781
Q9CR98	-0.05938	0.35764
E9QPX3	0.08052	0.35591
Q8R104	-0.15210	0.35571
Q9Z1P6	0.08486	0.35496
P56383	-0.18512	0.35364

Accession	Fold Change	-LOG(P-Value)
Q8R035	-0.08869	0.34481
P56375	-0.06296	0.34299
Q9D0K2	0.06077	0.34288
Q499X9	-0.09812	0.34064
Q8K215	0.09228	0.33993
P47791	0.09272	0.33877
Q8BJU9	-0.05372	0.33456
Q8BWT1	0.07125	0.33173
P56213	-0.07118	0.33081
Q3U8Y1	0.05846	0.33079
Q9Z0X1	0.04374	0.33064
P32020	0.06293	0.32868
E9PZS5	-0.12242	0.32776
P62264	-0.06427	0.32695
Q922B1	0.08725	0.32507
Q8K2C6	0.05508	0.32500
Q6P3A8	0.03438	0.32420
E9QJW0	0.05297	0.32391
Q8R2K3	-0.06258	0.32266
Q91VT4	0.09426	0.32062
Q91VM9	0.04688	0.31972
Q9CRD0	0.07835	0.31855
Q9D8H7	0.05066	0.31600
Q9CZU6	0.07707	0.31385
P53395	0.04960	0.30795
Q8BK30	0.16263	0.30690
Q9D7N6	-0.06126	0.30498
Q3U422	0.06634	0.30384
Q9CQL4	-0.06334	0.30307
O55126	0.05002	0.29829
Q9QX60	-0.06781	0.29241
Q99N95	0.11217	0.29113
Q8R0Y8	0.07426	0.29038
Q8CAK1	0.03832	0.28594
Q99KK9	0.05486	0.28468
P35290	0.12450	0.27951
P51174	0.04437	0.27863
P35487	-0.04707	0.27816
Q922E6	0.08377	0.27684
Q8VE38	-0.04114	0.27621
O88441	0.05741	0.27520
Q99JT1	0.08943	0.27282
Q9JKF7	-0.03710	0.27171
Q8BVU5	-0.07136	0.26966
O88696	0.03417	0.26828
Q9WTP7	0.03356	0.26603
Q9WVL0	0.07125	0.25559
P0DJF2	-0.05258	0.25533
P42125	0.04541	0.25349
Q8BGB8	-0.03363	0.25289
Q9DCI9	0.05276	0.25242
Q9DCJ5	0.06164	0.25241

Accession	Fold Change	-LOG(P-Value)
Q9CPY1	0.10860	0.25111
Q6P8J7	0.04625	0.25012
P12787	0.04625	0.24960
O55003	0.11506	0.24080
Q8R086	0.04813	0.23937
Q9DCV4	-0.10030	0.23819
E9Q1L7	-0.03169	0.23487
Q8R1S0	0.05592	0.23323
Q922Q4	-0.10338	0.23218
Q6RUT7	-0.07247	0.23218
Q9CXT8	0.03356	0.23180
Q9R0H0	0.05550	0.22542
F2Z456	-0.02274	0.22476
Q9CXW2	0.05783	0.22436
Q78IK4	0.05804	0.22385
Q3URS9	0.05023	0.22322
Q99MR8	0.02470	0.22178
P47738	0.02778	0.22073
Q9DC61	-0.06932	0.22065
Q921H8	0.03582	0.21686
Q9CRB8	-0.02761	0.21617
Q9CQF0	0.03895	0.21540
Q9D1H6	-0.03382	0.21289
Q5RKZ7	0.07987	0.21152
P52503	0.03852	0.21106
Q9JI39	0.14592	0.20994
Q9CQV1	0.03812	0.20932
Q3TLP5	0.05782	0.20310
G3X983	-0.05694	0.20255
Q8VEA4	-0.09034	0.20099
Q9QZD8	-0.07286	0.19953
P53702	-0.02450	0.19791
Q99J39	-0.02313	0.19787
O35435	0.03293	0.19784
Q3TC33	-0.09068	0.19609
Q9CQV5	-0.03285	0.19495
B7ZMP1	-0.06801	0.19461
Q80YD1	0.03459	0.19291
A6H611	0.07254	0.19209
P36552	-0.03053	0.18995
Q8R2Y8	0.05107	0.18898
Q71RI9	0.03479	0.18867
Q9CQ92	-0.03111	0.18769
Q9D773	0.03004	0.18729
Q8BP92	-0.03343	0.18668
Q9CXJ1	-0.03247	0.18481
Q5U5I3	-0.05239	0.18414
P19783	0.03521	0.18316
Q80ZS3	-0.03033	0.18010
P54071	0.02265	0.17903
Q5IRJ6-2	0.04498	0.17661

Accession	Fold Change	-LOG(P-Value)
P52825	0.02470	0.17616
Q8R5L1	0.02000	0.17591
Q8BWF0	-0.03343	0.17381
Q8C0K5	0.03791	0.17213
S4R2K0	0.02491	0.17180
P56379	0.05465	0.17086
P97493	0.03811	0.17083
Q9D338	0.07232	0.16966
O35465-2	-0.04936	0.16930
Q9CPQ1	0.04688	0.16900
Q9JLT4	0.03625	0.16752
Q9CPW3	-0.08154	0.16510
P34914	0.05382	0.16487
Q78J03	-0.02059	0.16325
Q8BIP0	0.03998	0.16238
Q9D8B6	0.03584	0.15795
Q9ERI6	0.17057	0.15734
Q9CRA7	-0.04056	0.15238
Q8BK08	0.03149	0.15169
Q3ULD5	0.02000	0.14586
Q91V61	0.01837	0.14569
Q9CZN8	0.02265	0.14520
Q8CEE7	-0.02489	0.14514
P53986	0.12200	0.14391
Q8VCX5-2	0.07426	0.14227
Q9DCM2	-0.03479	0.14122
Q9DCU6	-0.03882	0.14119
Q91ZE0	-0.02216	0.13493
Q8BYM8	-0.03169	0.13295
Q9QYA2	0.01410	0.12802
Q9ESW4	0.03314	0.12711
P03899	0.04520	0.12692
Q8BXN7	-0.02352	0.12668
Q9CQZ6	0.02531	0.12542
Q9CPV4	-0.01252	0.12317
Q9D7J9	-0.03845	0.12170
F7C846	0.02470	0.11817
Q8BGD8	-0.03033	0.11753
Q9CY16	0.02881	0.11733
Q8JZU2	-0.03169	0.11623
Q8R3F5	-0.02819	0.11426
Q9D7K5	-0.05447	0.11426
Q9DBF1	0.01939	0.11419
D3Z7P3	-0.01941	0.10799
Q9CR76	-0.04744	0.10711
Q8BUY5	0.03542	0.10626
Q99N92	-0.01351	0.10563
Q9D7N3	-0.02645	0.10523
Q80X85	0.01837	0.10447
Q3U186	-0.01666	0.10366
Q61578	-0.02586	0.10255
H3BLL2	-0.02956	0.09981

Accession	Fold Change	-LOG(P-Value)
Q9D2R8	-0.01370	0.09569
Q8BHC4	0.02143	0.09517
Q8K4F5	0.02347	0.09507
Q5XJY4	-0.04915	0.09504
Q9D6T9	0.02696	0.09327
P00397	-0.02567	0.09240
Q64133	-0.01587	0.09208
P51175	0.03355	0.09199
Q8K126	0.07426	0.08784
M0QWX7	-0.04267	0.08721
Q8JZM0	0.01066	0.08160
Q9JIY5	0.01086	0.08150
Q3TL44	0.02000	0.07803
Q9CQX8	-0.01450	0.07602
Q8K1R3	0.02347	0.07440
P51660	0.00944	0.07420
Q14DH7	-0.03169	0.07266
Q9CXD6	0.01309	0.07195
Q9WUR9	0.01613	0.07140
Q3UIU2	0.01025	0.06848
E9PZS8	0.02000	0.06363
K3W4M4	0.00743	0.06355
Q9DCJ7	-0.01587	0.06324
Q9Z0V7	-0.01173	0.06289
Q4VWZ5	-0.01153	0.06050
P60603	0.03687	0.06019
Q9CQA6	0.01592	0.05989
Q99LB2	-0.01193	0.05975
Q91WK5	0.01228	0.05881
P56393	-0.03053	0.05784
Q9D8S4	-0.01035	0.05729
P08228	0.01471	0.05718
Q9WUR2	0.00481	0.05548
Q8VCM5	0.02984	0.05361
Q9D0I9	0.02184	0.05322
D3Z7Q2	-0.01882	0.05058
Q61102	0.02881	0.04733
Q9D1H8	-0.00975	0.04689
Q59J78	-0.01469	0.04543
Q99JY0	0.01045	0.04528
Q7TMF3	-0.00876	0.04280
Q9D6S7	-0.00876	0.04273
Q9JKL4	0.00642	0.04225
Q8CCM6-2	-0.00519	0.04128
P63030	-0.01193	0.03905
Q91VN4	-0.00538	0.03883
Q8K0C8	-0.01351	0.03784
Q8K3J1	-0.01193	0.03773
Q91WK1	-0.00578	0.03658
A2APY7	0.00803	0.03597
Q9CZD5	-0.01351	0.03564
Q91YJ5	0.00985	0.03055

Accession	Fold Change	-LOG(P-Value)
O08600	-0.00618	0.02910
Q9CPQ8	-0.00538	0.02832
O35459	-0.00379	0.02813
Q5SUD5	-0.00698	0.02515
Q3UQ84	-0.00578	0.02514
Q5SSK3	0.00381	0.02508
Q9D6K8	0.00521	0.02333
Q9DAT5	0.00481	0.02281
E9Q3H6	0.00541	0.02248
P43023	0.00381	0.01977
Q8BU88	0.00602	0.01961
Q8R0N6	-0.00240	0.01915
Q8VDC0	-0.00280	0.01902
Q9DB10	0.00723	0.01878
Q91Z53	0.00220	0.01472
Q8JZU0	0.00280	0.01228
Q2TPA8	-0.00180	0.00897
Q99JR6	0.00100	0.00879
P48771	0.00120	0.00785
Q9D9V3	-0.00339	0.00776
Q9D0L4	0.00321	0.00756
O09174	0.00120	0.00599
P06745	-0.00080	0.00519
Q9D1B9	-0.00080	0.00364

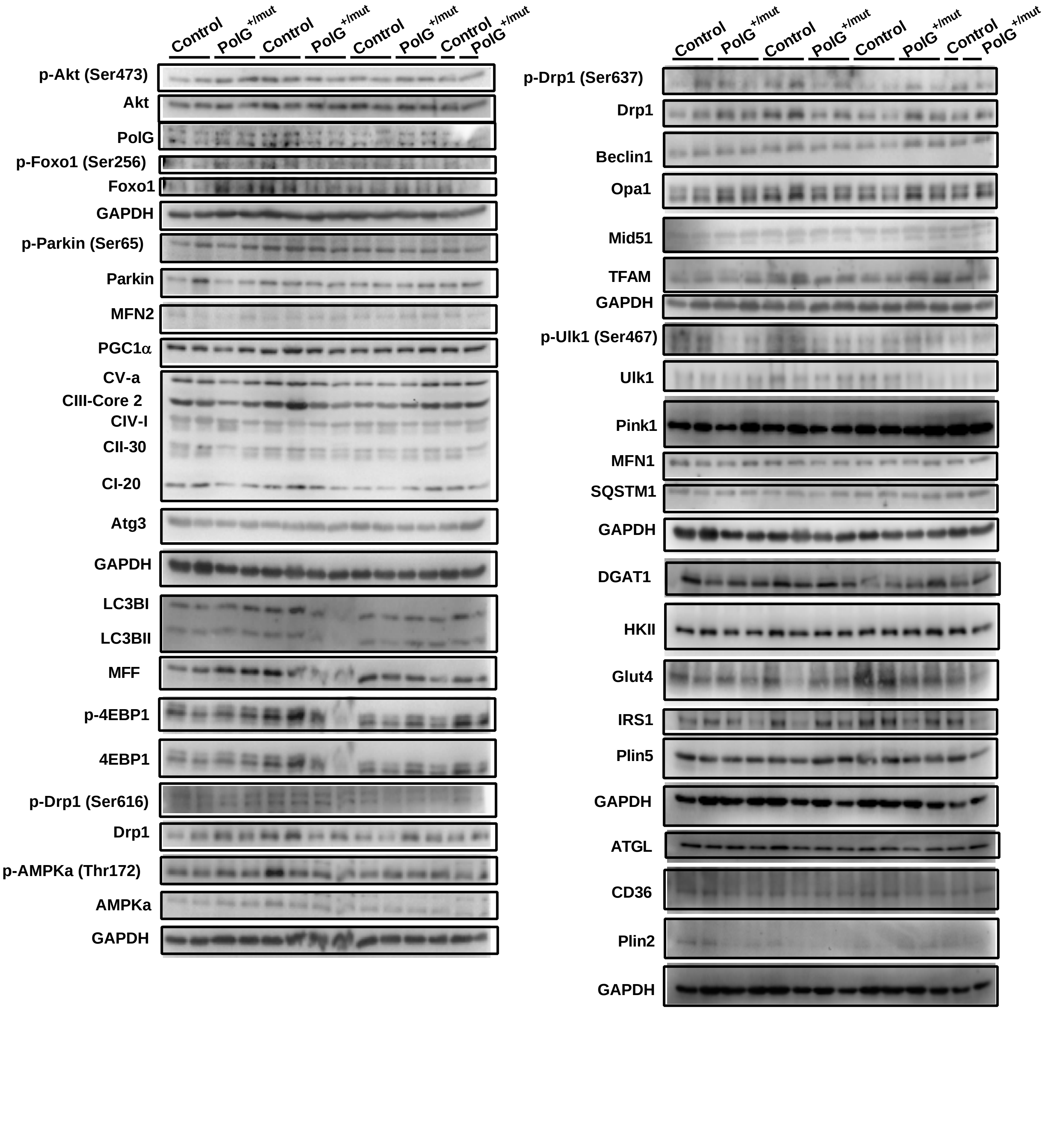

Supplemental Figure 1. Moore et al.

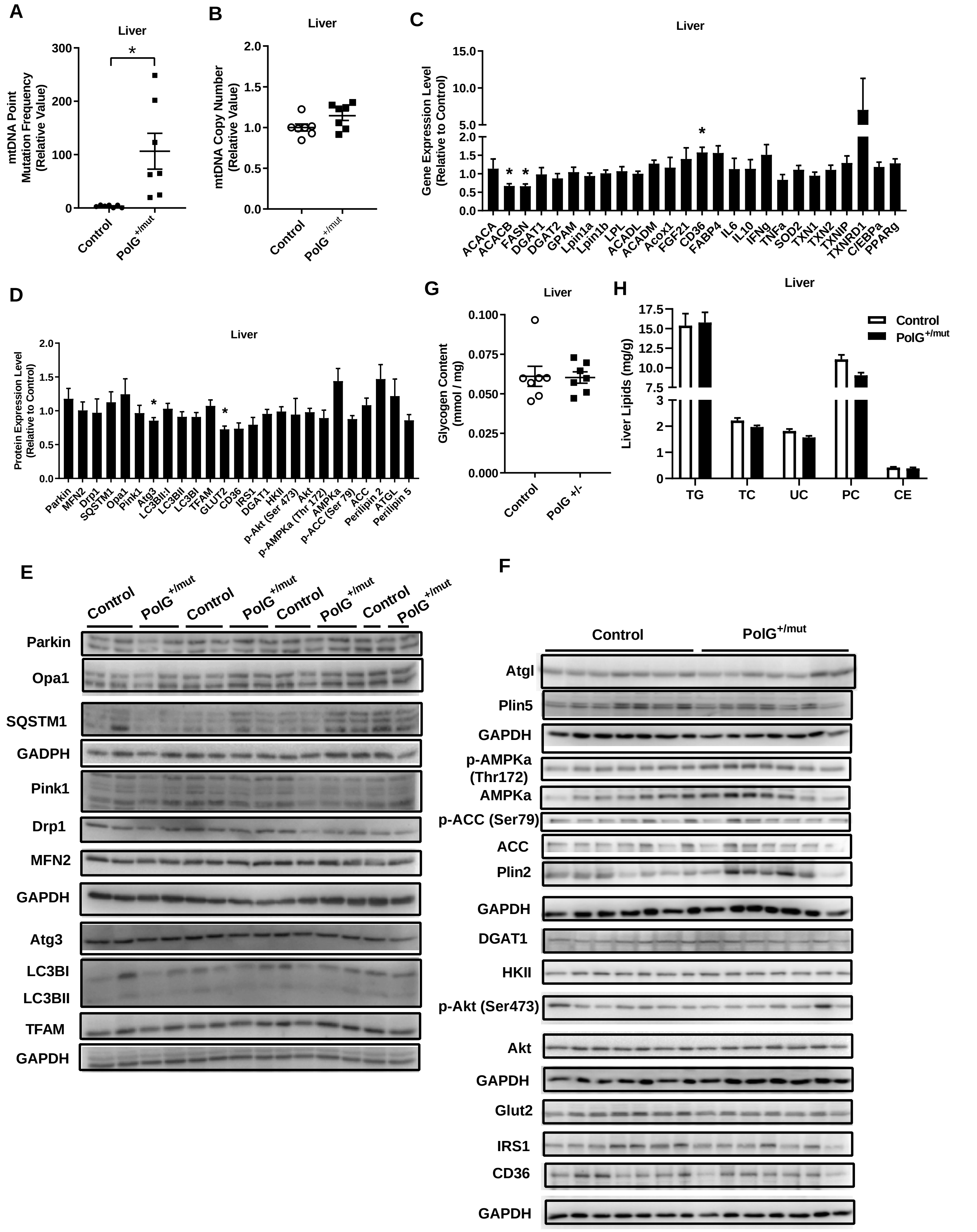

Supplemental Figure 2. Moore et al.

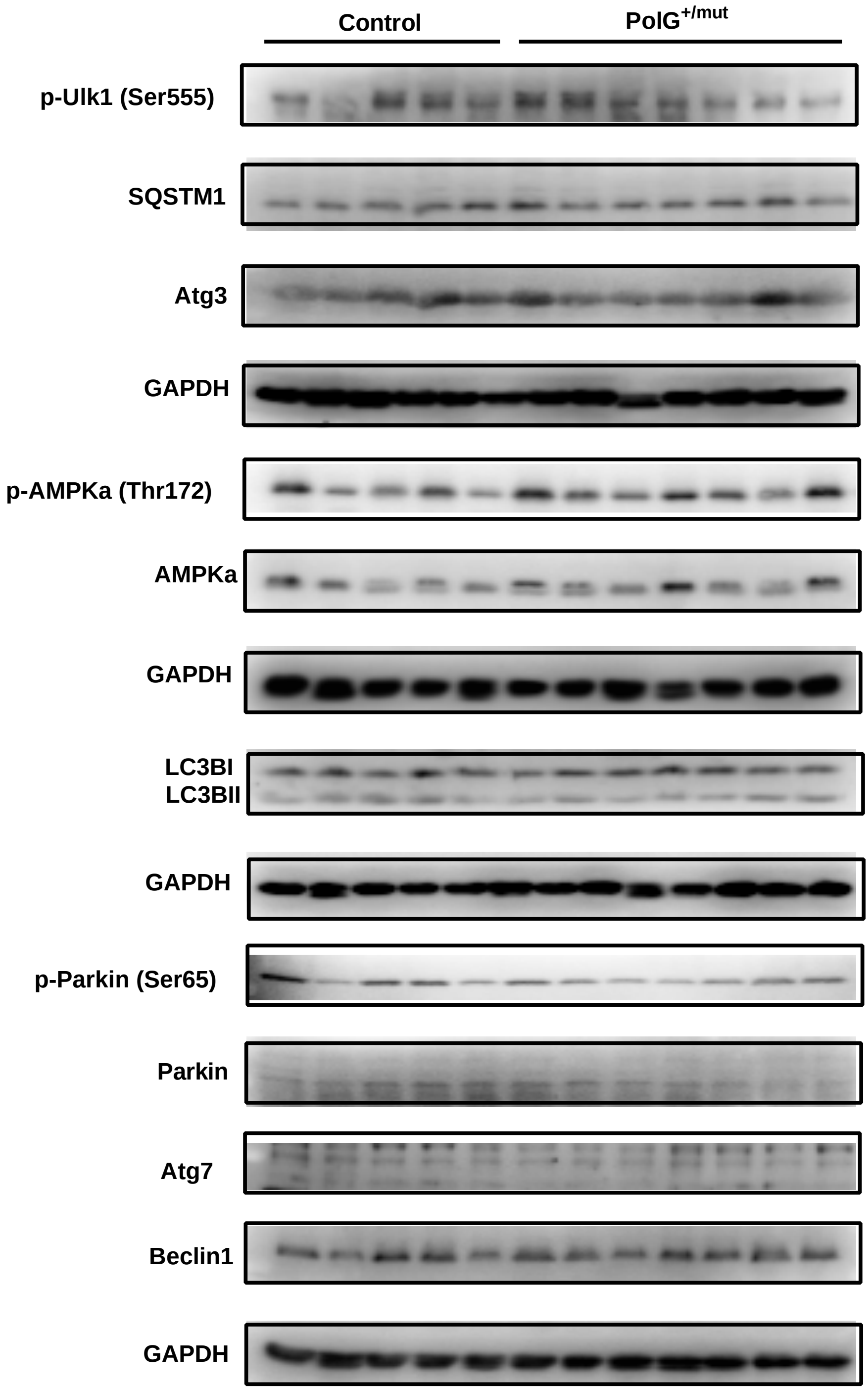

Supplemental Figure 3. Moore et al.

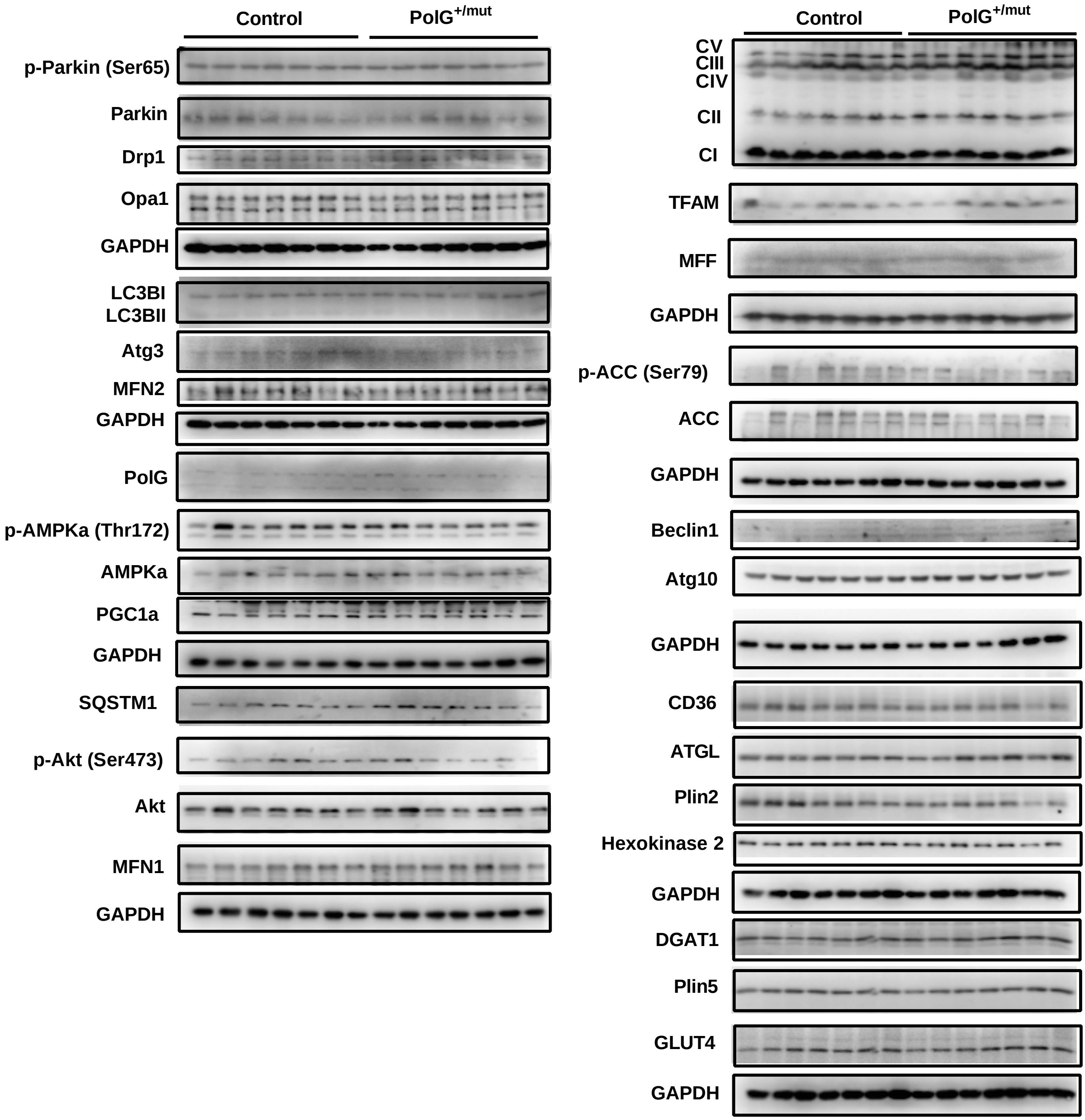

Supplemental Figure 4. Moore et al.

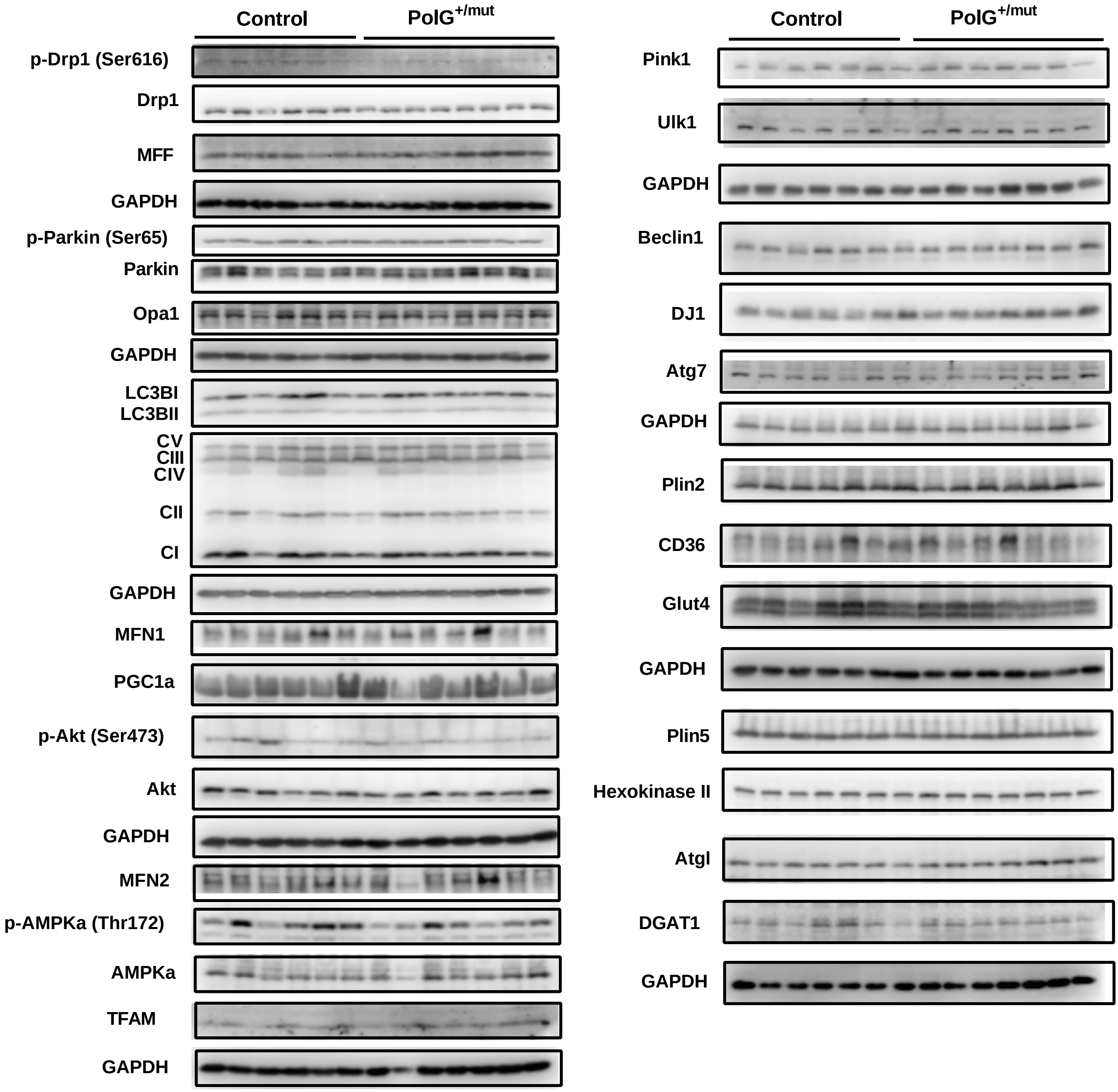

Supplemental Figure 5. Moore et al.
